# Non-reciprocal coalescence-breakup dynamics in flowing concentrated emulsions

**DOI:** 10.1038/s41467-026-75077-7

**Published:** 2026-07-06

**Authors:** Ivan Girotto, Andrea Scagliarini, Lei Yi, Roberto Benzi, Chao Sun, Federico Toschi

**Affiliations:** 1 The Abdus Salam, International Centre for Theoretical Physics, Trieste, Italy; 2 CNR-IAC, Rome, Italy; 3 INFN, Sezione di Roma Tor Vergata, Rome, Italy; 4 Department of Physics, University of Massachusetts, Amherst, MA USA; 5 Department of Energy and Power Engineering, New Cornerstone Science Laboratory, Center for Combustion Energy, Key Laboratory for Thermal Science and Power Engineering of MoE, Tsinghua University, Beijing, China; 6 Sino-Europe Complex Science Center, School of Mathematics, North University of China, Shanxi, Taiyuan, China; 7 Department of Physics and INFN, University of Rome Tor Vergata, Rome, Italy; 8 Department of Engineering Mechanics, School of Aerospace Engineering, Tsinghua University, Beijing, China; 9 Physics of Fluids Group, Max Planck-University of Twente Centre for Complex Fluid Dynamics, University of Twente, Enschede, The Netherlands; 10 Department of Applied Physics and Science Education, Eindhoven University of Technology, Eindhoven, The Netherlands

**Keywords:** Fluid dynamics, Statistical physics, thermodynamics and nonlinear dynamics

## Abstract

Dense stabilized emulsions are mixtures of immiscible fluids where the high-volume fraction droplet dispersed phase is stabilized against coalescence by steric interactions. The production of emulsions-a key process in food, cosmetics and chemical industries-involves high-shear flows, elastic and steric interactions, and proceeds thanks to coalescence and breakup of droplets and interfaces. The complex interplay between all these interactions is key in determining both small-scale droplet morphology as well as large-scale emulsion rheology. It is well known that at a critical volume fraction, *ϕ*_*c*_, the emulsion loses stability, undergoing an extremely rapid process where the fluid components in the emulsion exchange roles. This process, called catastrophic phase inversion, which resembles in several respects a dynamical phase transition, has remained widely elusive from an experimental and theoretical point of view. In this work, we present state-of-the-art experimental and numerical data to support a dynamical-system framework capable of precisely highlighting the dynamics occurring in the system as it approaches the catastrophic phase inversion. Our study clearly highlights that at high volume fractions, the droplet population starts to fluctuate wildly, leading to dramatic changes in the emulsion’s rheology and stability. Additionally, we show that at approaching the critical volume fractions, the dynamics can be simplified as being controlled by the evolution of a correlation length represented, in our systems, by the size of the largest droplet. This dynamics shares a close connection with non-reciprocal phase transitions where two different physical mechanisms, coalescences and breakups, can get out of balance leading to large non-symmetric periodic excursions in phase space. We clarify the phenomenology observed and quantitatively explain the essential aspects of the highly complex dynamics of flowing stabilized emulsions undergoing catastrophic phase inversion. More generally, our approach sets the basis for the definition and modeling of a vast number of dynamical phase transitions in hydrodynamic systems out-of-equilibrium where the flow, or other advection mechanisms, can enhance both aggregation and breakup of aggregates.

## Abstract (web summary)

The dynamics of flowing concentrated emulsions, which are crucial in various industries, can lead to catastrophic phase inversion at high volume fractions. This study successfully combines experimental, numerical and theoretical approaches to elucidate the non-reciprocal coalescence-breakup dynamics in these emulsions, revealing critical fluctuations.

## Introduction

Many natural systems, made of basic entities suspended in a flowing fluid, display the tendency of forming dynamically evolving aggregates. The fluid flow plays a dual role: it can considerably accelerate the aggregation process and, at the same time, be responsible for the breakup of larger aggregates. At a critical concentration, this aggregation/breakup balance is broken and the system undergoes a phase transition with the formation of a single integral-scale aggregate. Transitions of this kind include the colloidal sol-gel transition in stirred vessels^1,2^, the clusterization dynamics of floaters on turbulent surfaces^3^, including the formation of micro- and macroplastics aggregates^4,5^, planetesimals formation where turbulence in the protoplanetary disk interferes with accretion^6^, collapse of foams^7,8^ and the catastrophic phase inversion (CPI) in flowing emulsions^9,10^. Here, we focus on the latter example, as a prototype of the universal phenomenon described above, that is amenable to controlled experimental and numerical investigation. At sufficiently high droplet volume fraction, emulsions are metastable and prone to phase inversion, namely a microstructural, irreversible, change towards the thermodynamically favored configuration, whereby the continuous minority phase becomes disperse (and vice versa droplets become the continuous matrix)^9,10^. When induced by increasing the concentration of dispersed phase, the phase inversion is sudden and abrupt, i.e., it is reminiscent of a catastrophic process, in the sense of the theory of catastrophes^11–13^. Despite some success as a fitting method for experimental data, the catastrophe theory approach to phase inversion suffers serious limitations, specifically for what concerns its predictive capability and the ambiguous physical interpretation of the various parameters^14,15^. Even more importantly, the complex flow environment (turbulent or chaotic) under which CPI in stirred emulsions takes place does not enter in the description. Yet, wildly fluctuating hydrodynamic stresses in the stirring process lead the emulsion out-of-equilibrium, tightly interwining mechanisms that include turbulent dispersion, viscous stresses, elasticity, hydrodynamics interactions with coalescence and breakup of droplets. As a matter of fact, CPI in forced systems still lacks a satisfactory theoretical interpretation. Here, leveraging state-of-the-art experiments and direct numerical simulations (DNS), we probe the rheological and morphological evolution of stirred emulsions. Two prototypical cases are considered: turbulent Taylor–Couette (TTC) flows (experiments) and homogeneous isotropic turbulence (DNS); these two types of flows are chosen precisely in view of their relevant differences (e.g., presence/absence of large scale shear flows and boundary layers), thus supporting the generality of our findings. Identifying the fraction of volume occupied by the phase-inverted emulsion as the relevant behavior (morphology) variable, we propose a stochastic dynamical system, built on kinetic grounds and able to quantitatively capture the statistical properties of the key empirical aspects. Experiments, simulations and the model provide agreeing evidence of clear signatures of criticality, namely: (i) the divergence of fluctuations as a finite value of the volume fraction is approached and (ii) the emergence of non-Gaussian, bimodal, probability distribution functions. In particular, the model pinpoints the role played by the intrinsic non-reciprocity of the breakup and coalescence processes, and by the flow-induced effective mechanical noise in driving the phase transition.

## Results

### Stirring emulsions at high volume fraction

Stirring an immiscible mixture generates a turbulent multiphase flow, whereby strong and fluctuating hydrodynamic stresses break larger droplets into smaller droplets. In turn, when approaching each other with sufficient kinetic energy to overcome disjoining pressure barriers and lubrication forces, droplets coalesce minimizing interfacial energy. This process leads to a dynamical equilibrium characterized by a statistically stationary distribution of droplet sizes (with a mean value typically compatible with the so-called Kolmogorov-Hinze size^16,17^). To achieve a concentrated (densely packed) emulsion of droplets (e.g., oil-in-water or O/W emulsion), a stabilization of the interface that inhibits coalescence is needed: in our experiments this is provided by surface-active impurities accumulated at the oil-water interfaces^18^, whereas in the simulations it is achieved through properly devised competing lattice interactions^19,20^. Moreover, it is necessary to slowly add the dispersed phase in order to maintain the system in an intrinsically metastable state, i.e., energetically less favored with respect to the inverted emulsion (i.e., water-in-oil, W/O). Further details on how this is realized in the experiments and in the simulations are given in the Materials and Methods section.

**Fig. 1:**
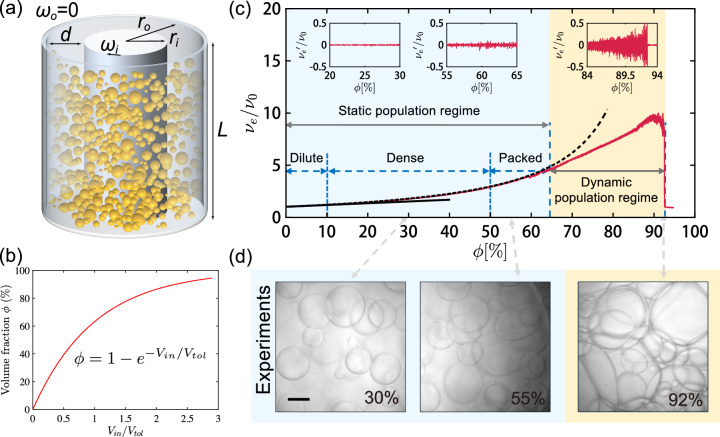
Experimental characterization of the emulsion under TTC. **a** A sketch of the experimental setup. The emulsion was maintained in the gap by rotating the inner cylinder at a constant angular velocity *ω*_*i*_ (see “METHODS”). Two micropumps were used to gradually change the volume fraction of the dispersed phase *ϕ*. **b** *ϕ* versus the volume of injected liquid *V*_in_/*V*_tol_. **c** For dilute volume fractions (*ϕ *≤ 10%), the effective viscosity *ν*_*e*_ increases almost linearly with *ϕ*, with droplets remaining well-separated and showing minimal direct interactions. In the dense regime, *ν*_*e*_ rises more steeply than linear as *ϕ* increases below 50%. When *ϕ* exceeds 50%, droplet deformation and enhanced *ν*_*e*_ are observed. The system remains in the static population regime for *ϕ* below  ~ 65%. For *ϕ* > 65%, the system transitions to a highly dynamic regime (dynamic population regime, DPR), marked by dramatically intensified fluctuations in the effective viscosity (${{\nu }_{e}}^{{\prime} }$ in the inset) and droplet size due to continuous coalescence and breakup among densely-packed droplets. The black solid line shows the Taylor relative viscosity *η*_*r*_ = 1 + 2.5*ϕ*(*λ* + 2/5)/(*λ* + 1) ^23^ (*λ* viscosity ratio (dispersed/continuous)), and the black dashed curve denotes the modified Krieger–Dougherty viscosity ${\eta }_{r}{\left(\frac{2{\eta }_{r}+5\lambda }{2+5\lambda }\right)}^{3/2}={\left(1-\phi /{\phi }_{m}\right)}^{-2.5{\phi }_{m}}$, where *η*_*r*_ = *ν*_*e*_/*ν*_0_ and the maximum packing fraction *ϕ*_*m*_ = *ϕ*_*c*_^24,48^. Here *Re* = 1.0 × 10^4^. **d** Experimental snapshots of emulsion at *ϕ* = 30% (dense regime), 55% (packed regime), and 92.5% (DPR). The scale bar is 200 μm. Here *Re* = 5.2 × 10^3^. Source data are provided as a Source Data file.

For low-to-moderate volume fractions of the disperse phase, it is customary to model the emulsification with population balance equations for the droplet size distributions^21^. Under these not too high concentration conditions, it is possible to attain a stable, weakly fluctuating, stationary droplet size distribution^22^; we call it, then, a static population regime. With reference to Fig. 1a, this corresponds to the region *ϕ* ≲ 65% (hereafter *ϕ* will denote the volume fraction of oil). In such static population regime, the experimental results reported in Fig. 1 show that, for dilute emulsions (*ϕ *≤ 10%), the system’s effective viscosity increases nearly linearly with *ϕ*, with droplets remaining well-separated and showing minimal direct interactions^23^. In experiments, the oil-in-water emulsion was produced in a Taylor–Couette flow system confined between two coaxial cylinders, where the outer cylinder is stationary while the inner cylinder rotates at a constant angular velocity to generate a turbulent emulsion (see Section “METHODS”). We gradually injected the dispersed phase into the system at a constant volume flow rate to slowly increase its volume fraction *ϕ* (a quasi-static process, see Section “METHODS” for details), and the instantaneous dispersed-phase volume fraction is given as a function of the injected liquid volume *V*_in_, i.e., $\phi=1-{e}^{-{V}_{{{\rm{in}}}}/{V}_{{{\rm{tol}}}}}$, where *V*_tol_ is the total volume of liquid in the system. For a given volume fraction *ϕ*, the effective viscosity of the emulsion system *ν*_*e*_ can be calculated as ${\nu }_{e}/{\nu }_{0}={(T/{T}_{0})}^{2.4}$, where *T* is the corresponding global torque *T* required to maintain a constant angular velocity (*ω*_*i*_) of the inner cylinder in our TC system, *ν*_0_ and *T*_0_ are the viscosity of the continuous phase (*ϕ* = 0%) and the torque of the system with *ϕ* = 0% at the same rotational angular velocity, respectively. This method is based on the assumption that, for various *ϕ* and *Re*, the momentum transport between the inner and outer cylinders in the TC system can be described by the same mechanism, of which the details can be found in Section “METHODS”. In dense conditions (10% ≲ *ϕ*≤50%), the effective viscosity rises more steeply than linear and above *ϕ* = 50%, one observes droplet deformation and enhanced effective viscosity. We have compared our viscosity data with the Taylor model for dilute emulsions (black solid line in Fig. 1c)^23^ and the modified Krieger–Dougherty (K–D) relation for concentrated emulsions (black dashed curve)^24^. The Taylor model only describes the viscosity evolution at low volume fractions (*ϕ* ≲ 10%). Interestingly, using the maximum packing fraction *ϕ*_*m*_ = *ϕ*_*c*_, the modified K–D model captures the experimental data very well until *ϕ* ≈ 65% (static population regime), after which the model overestimates the viscosity in the dynamic population regime where the droplet breakup and coalescence plays an important role. In addition, it should be noted that our effective viscosity is measured in a turbulent emulsion flow, which differs from the laminar flow conditions under which these classical shear-viscosity models are formulated. When *ϕ* > 65%, the system transitions to a highly dynamic population regime characterized by dramatically intensified fluctuations in both effective viscosity (see top insets of Fig. 1) and droplet size, resulting from continuous coalescence and breakup events among densely-packed droplets. The value of volume fraction (*ϕ* ≈ 65%) discriminating between static and dynamic population regimes corresponds, approximately, to the random close packing of spheres in three dimensions. Moreover, if we define the characteristic droplet ’lifetime’ as the mean interval of time between two consecutive breakup/coalescence events involving the same droplet, which can be estimated as *t*_*D*_ = 〈*N*〉/〈*β*〉 (where 〈*N*〉 is the mean number of droplets and 〈*β*〉 the mean breakup rate), it turns out from our numerical simulations (see Fig. 3 and Table 1 in ref. ^25^), interestingly, that *ϕ* ≈ 65% corresponds, approximately, to the volume fraction at which *t*_*D*_ becomes of the order of the large eddy turnover time, suggesting that the hydrodynamic flows and the interfacial dynamics start to get tightly coupled. While the dynamic population regime exhibits, as expected, higher effective viscosity (Fig. 1c), and consequently lower effective Reynolds number and thus lower turbulent fluctuations, we surprisingly find extreme fluctuations in both the effective viscosity (insets in Fig. 1c) and droplet size variations from experiments (Fig. 1d). At a critical volume fraction *ϕ*_*c*_, in the dynamic population regime, the metastable emulsion undergoes a catastrophic phase inversion, suddenly passing from a concentrated oil-in-water dispersion to a dilute water-in-oil dispersion (O/W  → W/O). Correspondingly, the effective viscosity drops abruptly to a low value, see Fig. 1a, compatible with that of a dilute emulsion at *ϕ*_*W*/*O*_ = 1 − *ϕ*_*c*_. It must be remarked that the actual value of *ϕ*_*c*_ depends on the flow conditions and on the physico-chemical properties of the interface (encoded in the dimensionless Reynolds, *Re* = *U*_rms_*L*/*ν*, and Weber, $We=\rho {U}_{\,{\mbox{rms}}\,}^{2}\langle D\rangle /\gamma$, numbers, where *U*_rms_ is the root mean square velocity, *L* is a characteristic large scale length, 〈*D*〉 is the mean droplet diameter, *ν* and *γ* are the kinematic viscosity and surface tension, respectively)^9,26–28^. Moreover, due to the diffuse interface nature of the numerical model, if a quantitative matching between experiments and simulations is sought for, a corrected effective volume fraction, taking into account the finite interface width, should be considered, as discussed in the Supplementary Material sections. Therefore, in what follows we will always refer to *ϕ*^(exp)^ and *ϕ*^(sim)^ for the volume fractions in experiments and DNS, respectively, whenever actual numerical values are involved. In TTC experiments, the Reynolds number can be estimated as *Re* = *ω*_*i*_*r*_*i*_*d*/*ν*_0_, where *r*_*i*_ and *d* are the radius of the inner cylinder and the gap width between the inner and outer cylinders. In our experiments, we have ${\phi }_{c}^{(\,{\mbox{exp}})}\approx 92.5\%$ at *Re* = 1.0 × 10^4^.

**Fig. 2:**
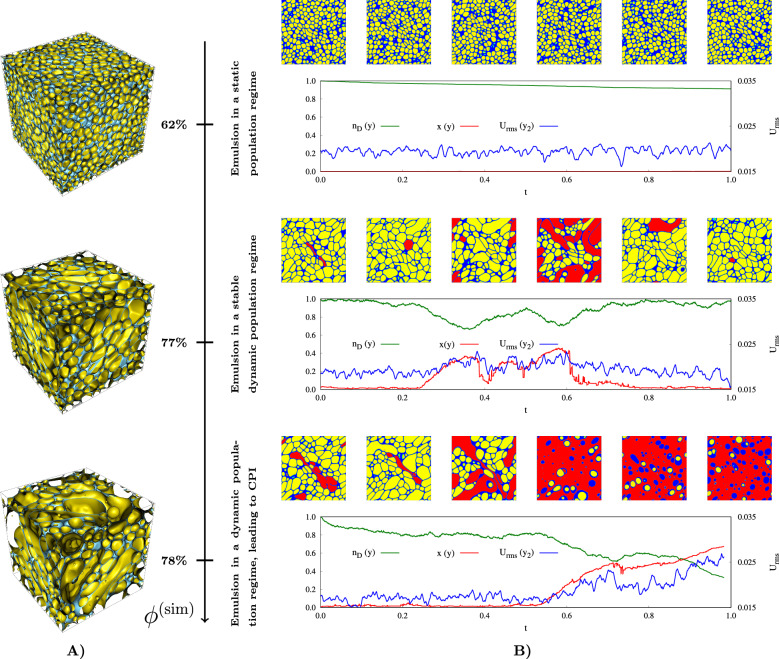
Population dynamics and CPI from DNS. **A** 3D view of density field snapshots at three different volume fraction of the dispersed (oil) phase, from top to bottom: *ϕ* = 62%, 77%, 78%. **B** For each volume fraction, 2D cross-sections (in the middle (*x*, *z*) plane) of the density field, taken every 2 × 10^5^ simulation steps along the time evolution, are shown. The region colored in red indicates the volume occupied by the largest droplet. In the graphs, the number of droplets (normalized by the maximum value over the whole run), ${n}_{D}(t)={N}_{D}(t)/{N}_{D}^{\,{\mbox{max}}\,}$, the fraction of total volume occupied by the largest droplet, *x*(*t*), and the mean square velocity, *U*_rms_(*t*), are reported as a function of time. The figures show negligible fluctuations for the packed emulsion, yet in the static population regime (*ϕ*^(sim)^ = 62%, top row), whereas a significant activity, with large amplitude excursions in the signals of *x*(*t*) and *n*_*D*_(*t*), is displayed when getting very close to the critical volume fraction (*ϕ*^(sim)^ = 77%, middle row. At increasing further the volume fraction (*ϕ*^(sim)^ = 78%, bottom row, the emulsion eventually and irreversibly loses stability and the catastrophic phase inversion takes place (as signaled by the simultaneous sudden growth of *x*(*t*) and drop of *n*_*D*_(*t*)). Source data are provided as a Source Data file.

Figure 2 helps us in getting insights on the emulsion morphology, whose evolution supports the distinction between the two population regimes below and above *ϕ*^(exp)^ ≈ 65% (arguably also this value, likewise *ϕ*_*c*_, is expected to be *Re*- and *We*-dependent). Numerical snapshots of the oil density field at various instants of times (see section “METHODS” for details), while the stirring force is active and the volume fraction is kept constant, are reported in Fig. 2 for, from top to bottom, *ϕ*^(sim)^ ≈ 62%, 77% and 78%. Oil regions are colored in yellow, water regions in blue, and the largest oil-connected region (the largest droplet) is colored in red. Each set of snapshots is shown together with the time evolution of the corresponding number of oil droplets (*N*_*D*_(*t*), divided by its initial value, *N*_*D*_(*t*_0_)), the volume of the largest oil droplet (normalized by the total volume), *x*(*t*), and the root mean square velocity (*U*_rms_). We observe that, for *ϕ*^(sim)^ = 62% (i.e., in the static population regime), both *N*_*D*_(*t*) and *x*(*t*) are almost constant in time, suggesting that droplet breakups/coalescences are unimportant. Accordingly, in this regime, also *U*_rms_ does not fluctuate significantly. At *ϕ*^(sim)^ = 77% (i.e., in the so-called dynamic population regime), instead, breakups and coalescences occur continuously in time, on average balancing each other and establishing a dynamical equilibrium. Such detailed balance can be occasionally and transiently broken giving rise to very large fluctuations where the largest droplet can occupy almost half of the total volume (*x*(*t*) ~ 0.4). These events of extreme transient growth and shrinkage of *x*(*t*) (excursions) come along with an important variation of the *U*_rms_, reflecting the fluidization of the part of the system that is locally phase-inverted. Catastrophic phase inversion occurs when the largest oil droplet invades the whole volume, eventually leading to the dispersion of water droplets in oil (see bottom row of Fig. 2, corresponding to *ϕ*^(sim)^ = 78%) and, correspondingly, *x*(*t*) ~ 1 − *ϕ*. The value of the critical volume fraction in the simulations can be, then, estimated as ${\phi }_{c}^{(\,{\mbox{sim}})}\approx 77.5\%$. The density maps in Fig. 2 also highlight that the inversion goes through states whereby regions of concentrated and phase-inverted emulsions coexist (double emulsions). We recall that phase coexistence in equilibrium systems is a trademark of discontinuous phase transitions^29^. The physics of the dynamic population regime-characterized by emulsion morphology and effective-viscosity fluctuations-is the central focus of this work and is discussed in detail in the following sections.

### Droplet population dynamics approaching CPI

**Fig. 3:**
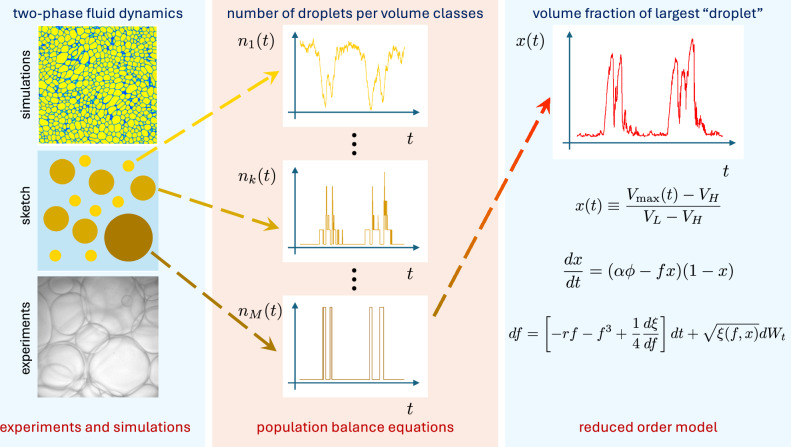
Concept map of the model reduction steps leading to the identification of *x*(*t*) (volume fraction occupied by the largest droplet) as the relevant order parameter describing the dynamics in proximity of the catastrophic phase inversion and its evolution equation (see Eq. (1)). (Left panel) All the complexity of dynamical evolution of the full 3D hydrodynamical interaction between droplets, including surface tension and disjoining pressure, eventually including breakup and coalescence events is illustrated. (Central panel) In the dynamic population description the system is effectively considered as zero-dimensional and the dynamics variables representing the number of droplets of a given size display a non-trivial temporal dynamics only when breakup and coalescence events occur, all the complexity of the visco-elastic and hydrodynamic physics are neglected. (Right panel) At approaching the CPI only the dynamics of the largest droplet is relevant in order to describe the rheology. This dynamics can be captured by a simple set of two non-reciprocal ODEs, for *x*(*t*) and for the breakup rate *f*, quantitatively modeling the statistics of the coalescence and breakup processes at varying the volume fraction *ϕ*.

The fraction of volume occupied by the largest oil droplet, *x*(*t*), features certain properties that make it a suitable candidate as a dynamical order parameter to probe the CPI. During the CPI transition, *x*(*t*) rapidly goes from a value close to zero to almost unity, furthermore for *ϕ* approaching *ϕ*_*c*_, the dynamics of *x*(*t*) develops large fluctuations (see Fig. 2), reminiscent of what was observed experimentally for the effective viscosity (Fig. 1). This suggests that *x*(*t*) may also work as a proxy for the rheological response of the material. In fact, in the proximity of the phase inversion and from a rheological point of view, the system can be seen as constituted of regions occupied by the concentrated emulsion (a yield-stress, non-Newtonian material with a relatively higher viscosity) and regions occupied by the phase-inverted emulsion (a Newtonian fluid with a relatively lower viscosity). The idea of identifying *x* as the relevant order parameter for the CPI transition is appealing, but deserves a more constructive justification. To this aim one may follow a conceptual path that echoes the usual model reduction approach leading from the atomistic view to the macroscopic continuum dynamics, which is pictorially represented in Fig. 3. The steady state of stirred non-dilute emulsions is a complex tangle of droplet deformation, breakup and coalescence, driven by hydrodynamic (turbulent) stresses, fluctuating in space and time; in a stable emulsion, on average breakup and coalescence balance, eventually determining a statistical distribution of droplet sizes peaked around a mean value (in general much smaller than the system size). From the perspective of emulsion morphology, this extremely complex spatiotemporal dynamics can be reduced to a set of balance equations of Smoluchowski type for droplet populations (probability distributions), *n*_*i*_(*t*) on the discrete space of size classes^21,30–32^ (see also the Supplementary Material for more details), as proven successful also for phenomena such as spray formation and, more in general, atomization of liquid jets and sheets^22,33,34^. With the increase of the volume fraction *ϕ*, the global rates of both coalescence and breakup show a steep growth, consistent with a diverging power-law function (as reported in ref. ^25^). The instantaneous imbalance of the two rates eventually leads to the emulsion destablization and CPI. We posit that, as we approach CPI, the kinetic-level description provided by the Smoluchowski approach might be further simplified, focusing only on the dynamics of the volume, *M*(*t*), of the largest droplet. It is the latter, in fact, that discriminates, by definition (it corresponds to the phase-inverted volume), when the phase-transition takes place. The whole hierarchy of equations for the various *n*_*i*_, with *i* < *M*, determines the effective in- and out-fluxes for the largest droplet, i.e., the growth or decrease rates for the dynamics of *x*.

### Non reciprocal phase transition

**Fig. 4:**
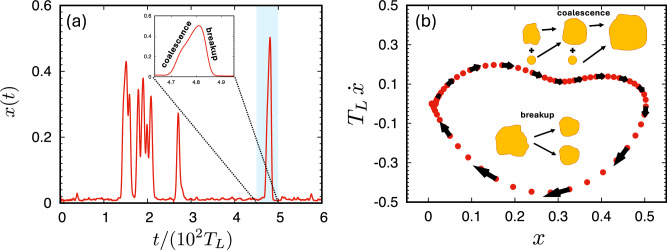
Large x fluctuations and breakup-coalescence non-reciprocity. **a** The signal of *x*(*t*) as a function of time, given in units of the characteristic large scale time *T*_*L*_ = *L*/*U*_rms_, from a simulation at *ϕ*^(sim)^ = 77%; in the inset we show the same signal restricted to the time interval which we report in (**b**). **b** Phase space portrait in the plane $(x,\dot{x})$ of the dynamics associated with a typical large-excursion, namely the one highlighted in (**a**) with a shaded area (and shown in the inset), plotted every 1600 simulation steps (corresponding to  ≈ 0.0625 *T*_*L*_); the arrows indicate the forward time evolution. Source data are provided as a Source Data file.

The dynamics of *x*(*t*) should be described by an ordinary differential equation featuring source and sink terms. The source term stems from the coalescence of smaller droplets (i.e., the input from the full population balance hierarchy), whereas the sink is due to the largest droplet breakup (into smaller droplets). A key empirical evidence regarding the largest droplet dynamics is that there is a fundamental asymmetry between coalescence and breakup: while coalescence-driven growth of *x* occurs by progressive absorption of small drops of normalized volume *δ* (*x* → *x* + *δ*), breakup is characterized by abrupt, mostly binary, events (*x* → *x*/2). This breakdown of detailed balance in the transitions between microscopic configurations causes the reported transient excursions in the signal of *x*(*t*) (Fig. 4a). The associated phase portrait, plotted in Fig. 4b, is represented by a closed curve in the $(x,\dot{x})$ plane, whose asymmetry with respect to the $\dot{x}=0$ axis confirms the microscopic detail balance breakdown and, consequently, suggests that the two processes, coalescence-driven growth and breakup-driven shrinkage of the largest droplet, are characterized by distinct timescales.

Since the coalescence rate is expected to be an increasing function of the volume fraction *ϕ*, a minimal dynamical model for *x* can be written down as:

1 $$\frac{dx(t)}{dt}=[\alpha \phi -f(t)x(t)][1-x(t)],$$

where *α* is a constant (a characteristic rate of coalescence), *f* can be interpreted as the breakup rate and the overall factor (1 − *x*) has been introduced since for *x* = 1 (i.e., the whole volume undergoes phase inversion) the process must stop. Figure 4a tells that the dynamics of *x*(*t*) displays bursting events where *x* reaches large values (*x* ~ 0.5), spaced out by relatively long periods where it remains small (*x* ~ 0). Modeling the ratio *α*/*f*, then, needs to take into account such observation. To this aim, we assume *α* to be constant and *f* to satisfy the stochastic differential equation:

2 $$df=\left[-rf-\beta {f}^{3}+\frac{1}{4}\frac{d\xi (f,x)}{df}\right]dt+\sqrt{\xi (f,x)}d{W}_{t},$$

where *W*_*t*_ is the standard Wiener’s process, i.e., it is a Gaussian process *δ*-correlated in time, and *r*^−1^ is a characteristic time for the fragmentation rate to vanish. The stochastic dynamics of *f*, as determined by the above equation, accounts for the effect of the fluid fluctuating turbulent stresses acting on the droplet interfaces and inducing them to break up. This effect is embedded in the variance of the multiplicative noise, $\xi (f,x)=\sqrt{{\varepsilon }_{0}+{\varepsilon }_{1}{f}^{2}(1-x)}$. Further details and theoretical justification of Eqs. (1)–(2) can be found in the Supplementary Material.

**Fig. 5:**
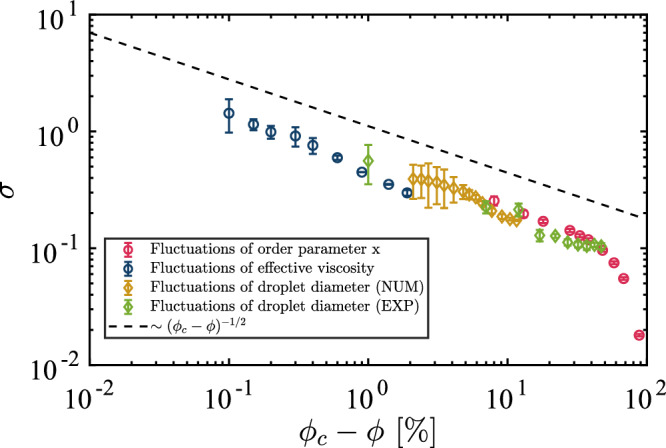
Fluctuations (standard deviation) of the order parameter, the effective viscosity, droplet diameter from experiments (EXP), and droplet diameter from numerics (NUM) as functions of (*ϕ*_*c*_ − *ϕ*), with ${\phi }_{c}^{(\,{\mbox{exp}})}=92.5\%$, ${\phi }_{c}^{(\,{\mbox{sim}})}=77.5\%$ and ${\phi }_{c}^{(\,{\mbox{mod}})}=98\%$ (for the model). Fluctuation data of the effective viscosity and droplet diameters (both NUM and EXP) are normalized with given constant values for comparison among different quantities here. The error bars depict the standard errors of the data (i.e., standard deviation divided by the square root of number of sample data). The dashed line highlights the power law divergence $\sigma \sim {({\phi }_{c}-\phi )}^{\chi }$, with *χ* = − 1/2. More precisely, a non-linear least square fitting of the function *f*(*x*) = *A* *x*^*χ*^ to the data in the range *ϕ* ≳ 60%, using a Levenberg-Marquardt algorithm, provides the following 90% confidence intervals for the exponents: *χ* = − 0.50 ± 0.04 (experiments), *χ* = − 0.48 ± 0.07 (numerics) and *χ* = − 0.50 ± 0.03 (model). Source data are provided as a Source Data file.

In Fig. 5 we plot the fluctuations of *x* (expressed by the standard deviation $\sigma=\sqrt{\langle {x}^{2}\rangle -{\langle x\rangle }^{2}}$, where the average is meant over time and over *O*(10^2^) realizations of the noise) obtained from the numerical integration of the model Eqs. (1)–(2), together with the effective viscosity fluctuations measured in the experiments and the fluctuations of the droplet diameters (from both experiments and simulations). In all cases, we observe a divergent behavior of *σ* as a function of the distance from the CPI transition point *ϕ*_*c*_ which appears consistent, within statistical errors, with the power law $\sigma \sim {({\phi }_{c}-\phi )}^{-1/2}$. It must be noted, as for any phase transition in a finite system, divergences will have a cutoff, specifically the variance of *x* cannot exceed unity and a real divergence would be expected only in the thermodynamic (infinite volume) limit. On the other hand, deviations from the power-law scaling at large *ϕ*_*c*_ − *ϕ*, i.e., far from the CPI, are not surprising, since scaling behavior is only to be expected near the critical point, even in standard equilibrium critical phenomena. Moreover, as we previously discussed, the very same approach in terms of the *x* variable should only be trusted as the CPI is approached, or, at least, in the close-packing regime.

Our study highlights a certain analogy between CPI and non-reciprocal phase transitions^35^, out-of-equilibrium transitions fundamentally distinct from equilibrium ones whose dynamics is governed by free energy minimization. In active matter, non-reciprocal systems exhibit time-dependent dynamical phases that induce chiral rotating phases and active time crystals absent in reciprocal systems^36^. These transitions unify non-Hermitian topology and active matter critical phenomena, describing collective behavior in systems that escape energy optimization principles. In our system, the asymmetric role of breakup and coalescence in the droplet population dynamics breaks microscopic detailed balance and, at the mathematical level, induces non-Hermitianity in the Jacobian of the deterministic limit (*W*_*t*_ = 0) of system (1)–(2)^35,37^.

### Bridging experiments, simulations and theory together: statistics of critical fluctuations

**Fig. 6:**
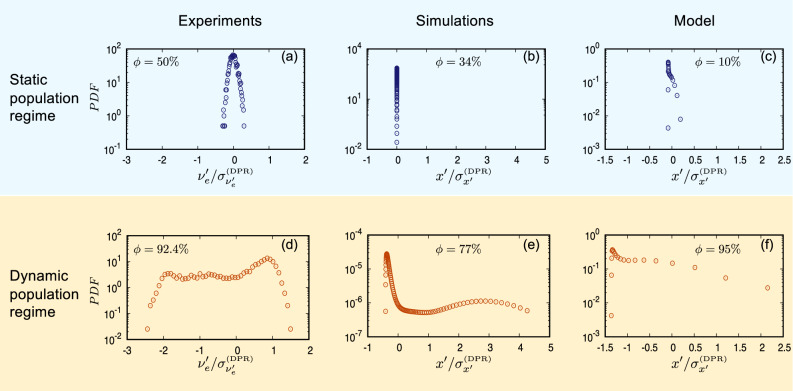
Probability density functions of fluctuations. PDFs of effective viscosity fluctuations ${{\nu }_{e}}^{{\prime} }$ from experiments (**a**, d), and PDFs of ${x}^{{\prime} }=x-\langle x\rangle$, fluctuations of the fraction of total volume occupied by the phase-inverted emulsion, from the direct numerical simulations (**b**, **e**) and from the model (**c**, **f**), at moderate volume fraction (in the ’static population regime’, (**a**–**c**)) and high volume fraction (in the ’dynamic population regime’, (**d**–**f**)). All quantities are given in units of their standard deviations in the dynamic population regime (${\sigma }_{{\nu }^{{\prime} }}^{({{\rm{DPR}}})}$ and ${\sigma }_{{x}^{{\prime} }}^{({{\rm{DPR}}})}$, respectively). Notice the bimodality in the dynamic population regime, with the secondary peak (at lower ${{\nu }_{e}}^{{\prime} }/{\nu }_{0}$, high *x*) originating from transient states where concentrated and phase-inverted emulsion coexist. Source data are provided as a Source Data file.

We measured the temporal fluctuations of the effective viscosity (applied torque) in the TTC experiment, as the volume fraction of initially dispersed phase is increased while keeping the angular velocity of the inner cylinder constant (further details can be found in “METHODS”). Close to the phase-inversion point, the average effective viscosity slightly decreases while the fluctuation is progressively growing. At some volume fractions, we fix *ϕ* and perform long-time measurements (step-by-step) to investigate the critical dynamics. Note that the step duration *τ*_exp_ = 60 min is ${{\mathcal{O}}}(1{0}^{6})$ times the turnover time scale of the flow. The most relevant aspect to be highlighted is that the probability density function (PDF) of the fluctuations of effective visocisty, ${{\nu }_{e}}^{{\prime} }$, which is Gaussian and narrow in the static population regime (dilute emulsion, Fig. 6a), tends to develop a non-Gaussian bimodal shape as the phase inversion is approached in the dynamic population regime (Fig. 6d). This suggests that the system spends most of the time in the concentrated emulsion state (main peak), but visits, with non-negligible frequency, the phase coexistence state discussed above (secondary peak at lower effective viscosity fluctuations ${\nu }_{e}^{{\prime} }$).

As previously discussed, we do expect the statistics of the effective viscosity, *ν*(*t*), to be closely related to that of the largest droplet volume, *x*(*t*). This is confirmed, indeed, in Fig. 6b, e, where we plot the PDFs of the fluctuations ${x}^{{\prime} }=x-\langle x\rangle$ obtained from the numerical simulations, in the two regimes. The PDFs share strong similarities with the experimental PDFs of ${{\nu }_{e}}^{{\prime} }$: notice, in particular, the presence, in the dynamic population regime, of the two peaks, with the secondary one associated to the occurrence of a partial phase inversion. Remarkably, the emergent bimodality as *ϕ* increases can be detected also in the PDFs of ${x}^{{\prime} }$ computed from the numerical integration of the model Eqs. (1)–(2) and reported in Fig. 6c, f.

## Discussion

**Fig. 7:**
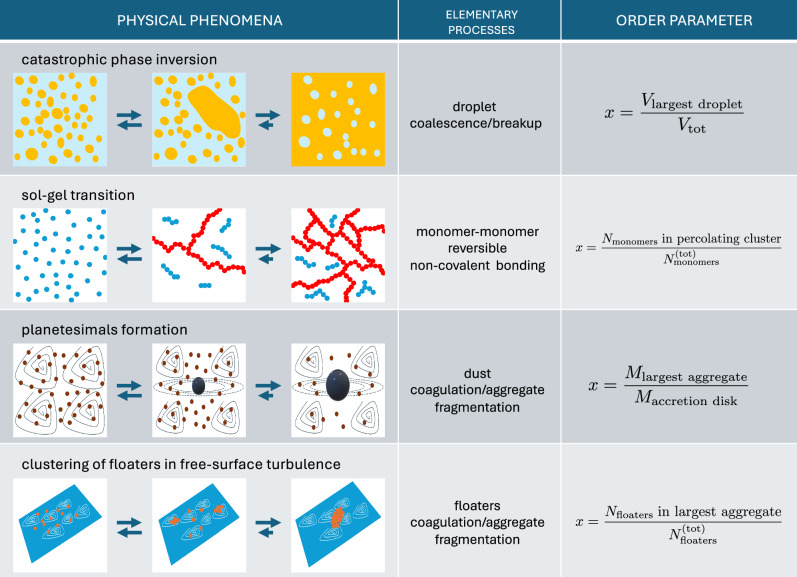
Some instances of physical systems whose dynamics is characterized by aggregation-fragmentation of elementary units in turbulent flow environments and such that a non-equilibrium phase transition with the formation of a single large scale aggregate can take place. From top to bottom: catastrophic phase inversion in emulsions; sol-gel transition in stirred vessels; planetesimal formation in a turbulent proto-planetary disk; clustering of floaters in free-surface turbulent flows (e.g., micro-/macro-plastics on the ocean surface).

We have presented a comprehensive study combining experiments, numerical simulations, and theoretical modeling, aimed at characterizing the structural and morphological evolution of flowing stabilized emulsions as the volume fraction of the dispersed phase is increased up to the critical point where catastrophic phase inversion occurs. Two different flow conditions have been considered: a Taylor–Couette setup (experiments) and large-scale homogeneous and isotropic forcing (simulations). We showed that below a volume fraction of  ≈ 65% (roughly corresponding to the random close packing of spheres in three dimensions), the droplet number and typical sizes do not fluctuate significantly; correspondingly, the effective viscosity of the system closely follows standard phenomenological correlations with the volume fraction (i.e., the Krieger–Dougherty law). Conversely, above 65%, strong deviations arise (attributable to the emergence of extremely non-Newtonian rheology), along with a markedly dynamic structural evolution in which breakup and coalescence play a key dynamical role, with their rates increasing dramatically^25^. Focusing on how the rheology changes as the system approaches catastrophic phase inversion, we introduced a novel theoretical framework characterized by the presence of an order parameter-the size of the largest droplet capable of bridging structural evolution and rheology through a common critical dynamics, appearing as a power-law divergence of fluctuations. Furthermore, we proposed a dynamical model able to reproduce the critical behavior of fluctuations of the order parameter while simultaneously highlighting the role of asymmetry and mismatched time scales between breakup and coalescence processes, revealing the close connection of catastrophic phase inversion with non-reciprocal phase transitions. Owing to the generality of the theoretical approach, our study offers a solid statistical physics framework to explain the basic physics beyond CPI in flowing emulsions, that -more generally- embraces all those systems undergoing a phase transitions characterized by broken detailed balance of small-scale aggregation-fragmentation processes (a few instances are reported and set in our theoretical framework in Fig. 7).

## Methods

### Experiments

The experiments are performed in a TTC system (Fig. 1a), which is the flow confined between two coaxial cylinders. The TC system is characterized by an outer cylinder of radius *r*_*o*_ = 35 mm, an inner cylinder of radius *r*_*i*_ = 25 mm, a gap *d* = *r*_*o*_ − *r*_*i*_ = 10 mm, and a height *L* = 75 mm. The emulsion confined between the two cylinders contains two immiscible liquids: silicone oil (density *ρ*_*o*_ = 866 kg/m^3^, viscosity *ν*_*o*_ = 2.1 × 10^−6^ m^2^/*s*) from Shin-Etsu and an aqueous ethanol-water mixture (75% volume fraction of water phase, *ρ*_*w*_ = 860 kg/m^3^, *ν*_*w*_ = 2.4 × 10^−6^ m^2^/*s*). Because the oil and ethanol-water mixture is inherently unstable, we maintain it in dynamic equilibrium using Taylor–Couette turbulent flow^38^, which continuously mixes the emulsion. This requires constant energy input through the rotation of the inner cylinder to sustain the turbulent state (the outer cylinder is stationary). A torque sensor was used to measure the torque *T* exerted on the inner cylinder with high accuracy, which is used to calculate the effective viscosity of the emulsion. Note that the densities of the two liquids are almost matched, eliminating the effect of the centrifugal force. The interfacial tension between the two immiscible liquids is *γ* = 5.7 ± 0.4 mN/m, which is measured using the pendant drop technique (oil droplet in ethanol-water) on a goniometer instrument (SCA20). No surfactant was intentionally added in the experiments. The closely-packed droplets in the dense emulsion before phase inversion require some kind of disjoining pressure for not fully coalescing. In our experiments, such a stabilizing effect could be sourced from unavoidable surface-active impurities accumulated at the oil-water interfaces, which can effectively suppress droplet coalescence. Possible sources of these surface-active impurities include trace contaminants in the working fluids (i.e., silicone oil and ethanol) and adsorption from the container walls, which has also been discussed in our previous work^18^. We stress that the reproducibility of the results is confirmed by 6 independent experiments. The droplet Weber number is given as $We=\rho \,{{U}_{{{\rm{rms}}}}}^{2}\,\langle D\rangle /\gamma$, where *ρ* is the density of the continuous phase, *U*_rms_ is the root mean square velocity, 〈*D*〉 is the average droplet diameter (of the order 〈*D*〉 ~ 200 − 400 *μ*m, see also Supplementary Fig. 1 in the Supplementary Material), and *γ* is the interfacial tension between the two phases. Based on the experimental measurements, the estimated *We* is around 2 to 5. In experiments, we gradually injected the dispersed phase (either oil or water) into the system and pumped out the well-mixed emulsion at the same constant volume flow rate *Q* to slowly increase its volume fraction *ϕ* (a quasi-static process), which is the continuous experiments (see Fig. 1). During injection, we determined the instantaneous oil volume fraction by assuming a uniform distribution of the injected liquid throughout the emulsion due to turbulent mixing. As the total volume of the liquid *V*_tol_ and the injection flow rate *Q* are known, the dispersed-phase volume fraction as a function of time (or injected liquid volume *V*_in_) can be analytically derived as:

3 $$\phi=1-{e}^{-\frac{{V}_{{{\rm{in}}}}}{{V}_{{{\rm{tol}}}}}}=1-{e}^{-\frac{Q}{{V}_{{{\rm{tol}}}}}t},$$

where *t* is the time from the start of injection. The exponential evolution of *ϕ* over *V*_in_/*V*_tol_ can be seen in Fig. 1b.

**Fig. 8:**
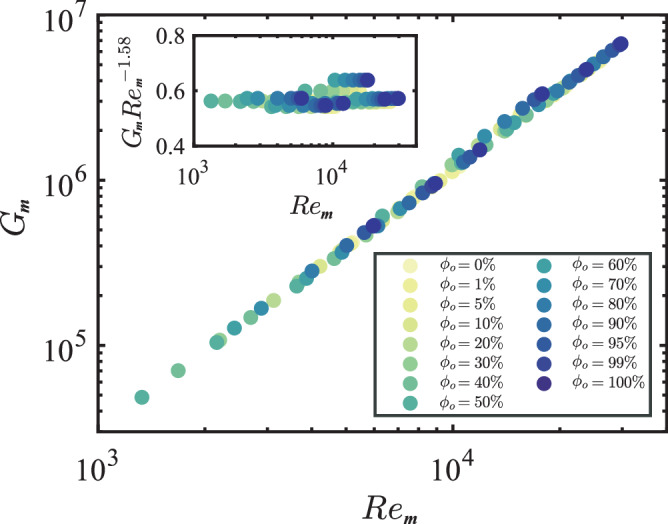
The dependence between the modified dimensionless torque *G*_*m*_ and the modified Reynolds number *Re*_*m*_, using the effective viscosity *ν*_*e*_. All these datasets at the various oil volume fractions *ϕ* collapse in a master curve. The inset denotes a compensated plot with ${G}_{m} \sim {R{e}_{m}}^{1.58}$. Source data are provided as a Source Data file.

We perform two types of measurements on the emulsion. The first measurement is the evolution of local droplet structures using a high-speed camera mounted with a microscopic lens, including their size and morphology (Fig. 1d). The second one is the system’s effective viscosity *ν*_*e*_, which is obtained through time-resolved global torque (*T*) that is required to maintain a constant angular velocity (*ω*_*i*_) of the inner cylinder in the TC system (Fig. 1(c)). Here we define the modified dimensionless torque as ${G}_{m}=T/(2\pi L\rho {{\nu }_{e}}^{2})$ and modified Reynolds number as *Re*_*m*_ = *ω*_*i*_*r*_*i*_*d*/*ν*_*e*_. For the pure ethanol–water mixture (*ϕ* = 0%) with a known viscosity *ν*_*e*_ = *ν*_0_, we observe a scaling law ${G}_{m} \sim R{e}_{m}^{1.58}$ (see Fig. 8). This gives ${G}_{m}=K{R{e}_{m}}^{1.58}$, where *K* is a constant prefactor. With the definitions of *G*_*m*_ and *Re*_*m*_, we obtain a dependence of the torque *T*_0_ on the viscosity *ν*_0_ as ${T}_{0}=AK{{\nu }_{0}}^{0.42}$ (*ϕ* = 0%), where $A=2\pi L\rho /{({\omega }_{i}rd)}^{0.42}$. This relation is expected to be valid for emulsions with various oil volume fractions *ϕ* and *Re*^28,39^. Thus, for a given *ϕ* and *Re*, the effective viscosity of the emulsion system *ν*_e_ can be calculated with the corresponding global torque *T* as

4 $$\frac{{\nu }_{{{\rm{e}}}}}{{\nu }_{0}}={\left(\frac{T}{{T}_{0}}\right)}^{2.4}.$$

We calculate *G*_*m*_ and *Re*_*m*_ using the effective viscosity obtained for each case, and find that all datasets at various *ϕ* collapse onto a master curve (see Fig. 8). More details on the calculation of the effective viscosity can be found in refs. ^18,40^. Additionally, we performed step-by-step experiments (see Figs. 5 and 6), measuring the torque at a fixed volume fraction *ϕ* for a duration *Δt* at each step, while *ϕ* is increased stepwise until the phase inversion occurs. Note that the step duration *Δt* = 60 min (or *Δt* = 20 min) is ${{\mathcal{O}}}(1{0}^{6})$ times the turnover time scale of the flow. We clarify that both the continuous experiments and step-by-step experiments yield reproducible results and lead to reliable conclusions. In particular, the step-by-step measurements supported include 6 independent experiments, including 5 experiments with a step duration of *Δt* = 20 min and 1 experiment with a step duration of *Δt* = 60 min. All repeated experiments yield consistent results and support the conclusions of the manuscript. The reproducibility is explicitly reflected in Fig. 5, where the fluctuations near the phase inversion are shown with error bars that quantify the variability across repeated experiments.

### Numerical simulations

The simulations are performed integrating numerically, by means of a two-component lattice Boltzmann method (LBM)^41,42^, the equations of motion for the two fluid density fields, indicated as *O* and *W* (for, e.g., oil and water), *ρ*_*O*,*W*_, and the incompressible barycentric fluid velocity field, **u**:

5 $${\partial }_{t}{\rho }_{\sigma }+\nabla \cdot ({\rho }_{\sigma }{{\bf{u}}})=	 0\quad \sigma=O,W \\ {\rho }_{f}({\partial }_{t}{{\bf{u}}}+{{\bf{u}}}\cdot \nabla {{\bf{u}}})=	 -\nabla p+\nabla \cdot {{{\bf{P}}}}^{{{\rm{(mix)}}}}+\eta {\nabla }^{2}{{\bf{u}}}+{{{\bf{F}}}}^{({{\mbox{ext}}})},$$

where *ρ*_*f*_ = *ρ*_*O*_ + *ρ*_*W*_ is the total density, *p* is the pressure, *η* is the dynamic viscosity and **F**^(ext)^ a forcing term. ${P}_{ij}^{(\,{\mbox{mix}})}[{\rho }_{O},{\rho }_{W},{\partial }_{i}{\rho }_{O},{\partial }_{i}{\rho }_{W},\ldots \,]$, a function of the two density fields and of their derivatives, is the non-ideal contribution to the pressure tensor. In this hydrodynamic framework, the surface tension, *γ*, is computed from its mechanical definition as the integral of the mismatch between the normal and tangential components of **P**^(mix)^ across a flat interface at equilibrium, i.e., considering without loss of generality a 2*d* problem, $\gamma=\int_{-\infty }^{+\infty }\left({P}_{xx}^{(\,{\mbox{mix}})}-{P}_{yy}^{({\mbox{mix}})}\right)(x)dx$^43^. Analogously, the disjoining pressure, *Π*, can be determined by measuring the overall film tension, *Γ*_*f*_, as the integral of the normal-transversal stress tensor mismatch across two flat interfaces separated by a liquid layer of width *h*. The disjoining pressure is related to *Γ*_*f*_ as $h\frac{d\Pi }{dh}=\frac{d{\Gamma }_{f}(h)}{dh}$^20,44^.

We remark that a fully hydrodynamic approach of this kind is justified here since we aim to describe emulsions made of droplets of sizes *d* ~ 10^2^ μm (see previous section), such that thermal fluctuations may be neglected, in fact, at room temperature *T* ≈ 300 K, $\sqrt{{k}_{B}T/\gamma }/d \sim {{\mathcal{O}}}(1{0}^{-6})$. The large-scale forcing needed to generate the chaotic stirring that mixes the two fluids, **F**^(ext)^, takes the following form:

6 $${F}_{i}^{\,{\mbox{(ext)}}\,}({{\bf{x}}},t)={\sum}_{\sigma }{F}_{\sigma i}^{\,{\mbox{(ext)}}\,}({{\bf{x}}},t)=A{\sum}_{\sigma }{\rho }_{\sigma }{\sum}_{k\le 2\pi \sqrt{2}/L}{\sum}_{j\ne i}\left[\sin ({k}_{j}{x}_{j}+{\varphi }_{j}^{(k)}(t))\right],$$

where *i*, *j* = 1, 2, 3, *A* is the forcing amplitude, **k** is the wavevector, and the sum is limited to ${k}^{2}={k}_{1}^{2}+{k}_{2}^{2}+{k}_{3}^{2}\le 8{\pi }^{2}/{L}^{2}$. The phases ${\varphi }_{j}^{(k)}$ are evolved in time according to independent Ornstein-Uhlenbeck processes with the same relaxation times *T*_*L*_ = *L*/*U*_rms_, where *L* is the cubic box edge and *U*_rms_ is a typical large-scale velocity^45,46^. All the data presented in this work come from simulations with *L* = 512.

## Supplementary information

Supplementary Information

Transparent Peer Review File

## Data Availability

Source data for this study have been deposited in the Zenodo database under accession code 10.5281/zenodo.20553861^47^.

## Code availability

The code executable, together with a sample input file and a text file specifying how the code can be run, is provided on Zenodo at 10.5281/zenodo.20553861^47^. The Fortran code integrating the model equations, Eqs. (1)-(2), together with a short explanatory text are also provided^47^.
