# Supplementary material for: Non-reciprocal coalescence-breakup dynamics in flowing concentrated emulsions
Source: Nat Commun. 2026 Jul 6;17:8339. doi: 10.1038/s41467-026-75077-7 (PMC13473571; doi:10.1038/s41467-026-75077-7)
Supplement: Supplementary file 1 [file 41467_2026_75077_MOESM1_ESM.pdf]

Supplementary Material for: "Non-reciprocal coalescence-breakup dynamics in flowing concentrated emulsions"

Ivan Girotto,[1,*] Andrea Scagliarini,[2,3,*] Lei Yi,[4,5,*] Roberto Benzi,[6,7,†] Chao Sun,[5,8,9,†] and Federico Toschi[10,2,†]

[1] *The Abdus Salam, International Centre for Theoretical Physics, Trieste, Italy*

[2] *CNR-IAC, I-00185 Rome, Italy*

[3] *INFN, Sezione di Roma Tor Vergata, I-00133 Rome, Italy*

[4] *Department of Physics, University of Massachusetts, Amherst, Massachusetts 01003, USA*

[5] *New Cornerstone Science Laboratory, Center for Combustion Energy, Key Laboratory for Thermal Science and Power Engineering of MoE, Department of Energy and Power Engineering, Tsinghua University, 100084 Beijing, China*

[6] *Sino-Europe Complex Science Center, School of Mathematics, North University of China, Shanxi 030051, Taiyuan, China*

[7] *Department of Physics and INFN, University of Rome Tor Vergata, Rome I-00133, Italy*

[8] *Department of Engineering Mechanics, School of Aerospace Engineering, Tsinghua University, Beijing 100084, China*

[9] *Physics of Fluids Group, Max Planck-University of Twente Centre for Complex Fluid Dynamics, University of Twente, 7500 AE Enschede, The Netherlands*

[10] *Department of Applied Physics and Science Education, Eindhoven University of Technology, 5600 MB Eindhoven, The Netherlands*

(Dated: June 16, 2026)

I. THE VOLUME FRACTION CORRECTION IN NUMERICAL SIMULATIONS

The critical volume fraction for CPI depends, in general, on many physical and phyico-chemical parameters (fluid densities and viscosities, type of surfactants, stirring speed, etc...). When comparing numerical simulations with experiments, further factors come into play,

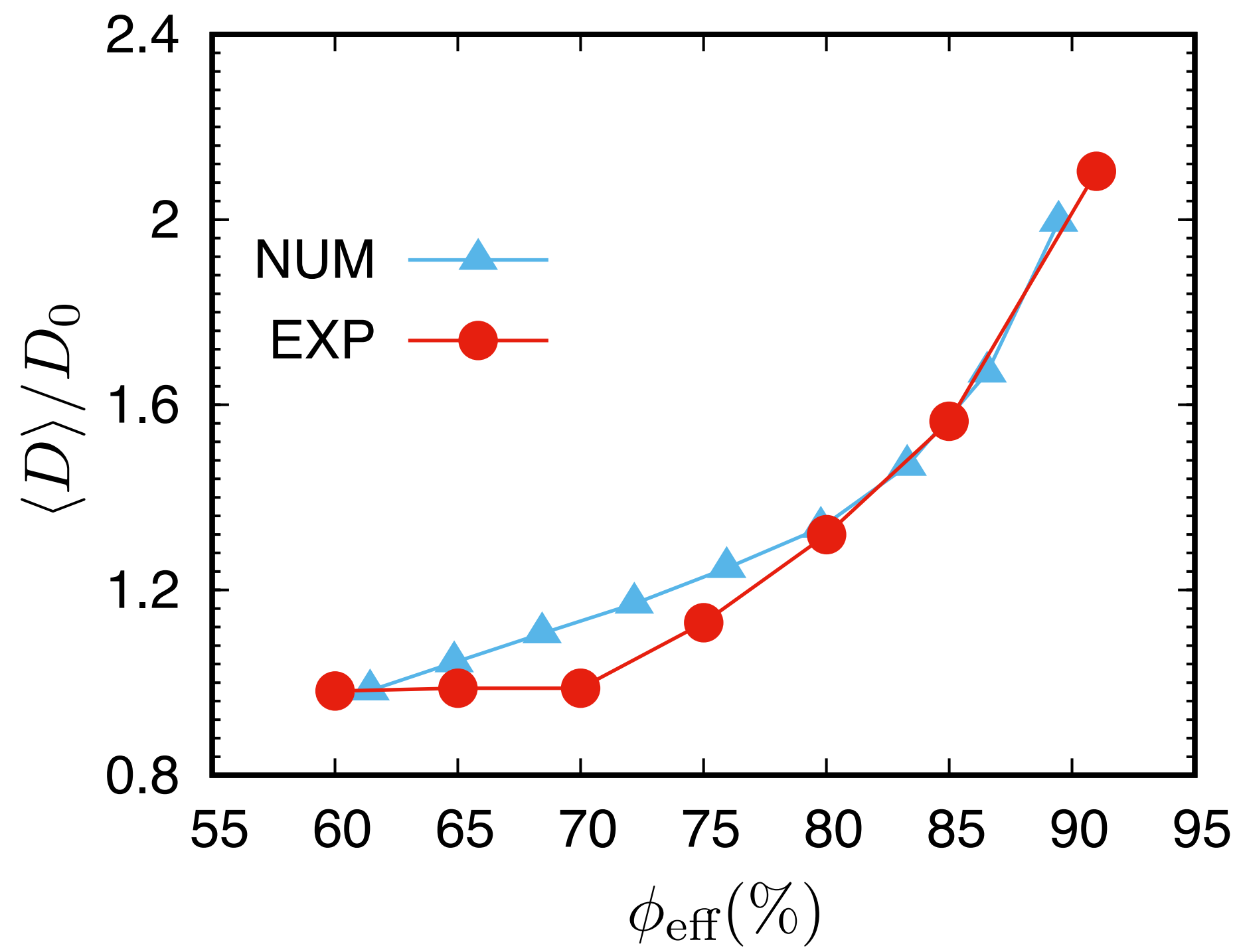

Supplementary Figure 1: Mean droplet diameter, $\langle D\rangle$ (normalized by D_0, the value at the lowest volume fraction), as a function of ϕ_{eff} from experiments (where $\phi_{\mathrm{eff}} \equiv \phi^{(\mathrm{exp})}$) and numerical simulations (in which case $\phi_{\mathrm{eff}} = \phi^{(\mathrm{sim})}(1+\delta/\langle D\rangle)^3$, $\delta = 3.0$ is the interface width). The value of D_0 in the experiment is $D_0 \approx 200\,\mu$m, whereas in the simulation it is $D_0 \approx 25\,$l.u. (lattice units). Source data are provided as a Source Data file.

since length and time scales differ. In our case, the diffuse interface nature of the numerical method (LBM) must be considered. Especially under closely packed conditions, in fact, the finite interface width, δ is relevant in the interaction between neighbouring droplets. To account for it, we attribute to each droplet (assumed spherical, for simplicity) an effective radius increased by $\delta/2$, such that the mean radius becomes $R + \delta/2$. This introduces

* Equally contributing authors

† Contact authors: FT: f.toschi@tue.nl; CS: chaosun@tsinghua.edu.cn; RB: roberto.benzi@gmail.com;

a correction to the volume fraction, that can be estimated as $\phi_{\text{eff}} = \frac{4\pi}{3V} N_D (R + \frac{\delta}{2})^3 = \frac{4\pi}{3V} N_D R^3 (1 + \frac{\delta}{2R})^3 = \phi(1 + \frac{\delta}{2R})^3$, where N_D is the number of droplets and $V = L^3$ the total volume. In Supplementary Figure 1 we report measurements of the mean droplet diameter, $\langle D \rangle$, from experiments and simulations, as a function of ϕ, rescaled, in the numerical case, by the factor $(1 + \delta/\langle D \rangle)^3$, with $\delta = 3$ lattice units; the data suggest that, indeed, the proposed correction allows for a reasonable one-to-one comparison. We finally note that, upon multiplication by the correcting factor, the critical volume fraction ($\phi_c^{(\text{sim})} = 77.5\%$) becomes $\phi_{c,\text{eff}}^{(\text{sim})} \approx 90\%$.

II. POPULATION BALANCE MODELS

To better highlight the morphological changes in the emulsion which become essential for the dynamics at approaching the critical volume fraction, we focus on a population description of the emulsion. In this approach, we disregard the space dimensionality and the hydrodynamics and elastic processes between droplets, to focus only on the dynamics of droplet sizes. The key description of the systems, which is considered effectively zero-dimensional, is the distribution of number of droplets $n_i(t) \equiv n(x_i, t)$ per size class, x_i, as a function of time. The size classes can be taken as geometrically distributed multiples of some characteristic droplet size (e.g. the Kolmogorov-Hinze size), or submultiple thereof, i.e. $x_i = x_{\text{KH}} \lambda^i$. Such type of processes are typically described in terms of a population balance equation (PBE), whose most generic form reads [1, 2]:

$$\dot{n}_i = B_i^{(b)} - D_i^{(b)} + B_i^{(c)} - D_i^{(c)} \quad \text{for } i = 1, 2, \ldots, M \tag{S1}$$

where $B_i^{(b,c)}$ and $D_i^{(b,c)}$ are birth and death rates of droplets in the i-th class due to breakup and coalescence, respectively. A more explicit instance of a PBE can be written as the following Smoluchowski-like coagulation-fragmentation equation [3, 4]:

$$\dot{n}_i = \sum_{j=1}^{M-1} p_j \beta_{i+j} n_{i+j} + p_i \sum_{j=i+1}^{M} \beta_j n_j - \left(\sum_{j=1}^{i-1} p_j \right) \beta_i n_i - \sum_{j=1}^{M-i} \kappa_{i,j} n_j n_i + \frac{1}{2} \sum_{j=1}^{i-1} \kappa_{j,i-j} n_j n_{i-j} \tag{S2}$$

The above equation provides a description of the time-dependent change of $n_i(t)$ due to the coalescence (with rates $\kappa_{i,j}$) and breakup (with rates β_i and probability p_j that in a breakup two droplets of size j and $i - j$ are formed). This Smoluchowski coagulation-fragmentation equation describes the change in sizes of droplets and the rates, $\kappa_{i,j}$ and β_i, are functions

of the underlying fluid dynamic processes that control the coalescence and breakup events; these include the Reynolds number of the flow, the surface tension, and disjoining pressure, as well as the volume fraction. From the fully resolved numerical simulation, the rates $\kappa_{i,j}$ and β_i can be accurately measured. A simplified representation of population dynamics as emerging from the simulations, in the dynamic regime, is reported in Supplementary Figure 2.

III. NON RECIPROCAL DYNAMICAL MODEL

In this section we further simplify the population model based on the Smoluchowski equation into a simpler model already discussed in the main part of the paper. The model aims at capturing the essential features of the dynamics in proximity of the critical volume fraction. The basic starting point is the consideration of the fact that at approaching the critical volume fraction the emulsion starts to develop larger regions of partially inverted emulsion. These regions, visible in Supplementary Figure 2, can be represented in terms of the volume of the largest droplets. We therefore assume that at approaching the critical volume fraction the Smoluchowski dynamics is actually controlled by the presence of a very large number of small droplets of size of the order of the Hinze radius, R_H and by one large droplet that, as a function of time, can grow, due to coalescence with smaller droplets, and shrink, due to breakup into smaller droplets. In these conditions the two dynamical variables essential to describe the dynamics are then the volume of the largest droplet, x, and the number of small droplets.

The key physical feature of the dynamics is the presence of two physically distinct phenomena, one associated to droplets coalescence and the other with droplets breakup. The typical timescales of the two processes are different, as well as their dependency on the underlying hydrodynamic processes. Next, we assume that, close to the phase inversion, the statistical property of the emulsion can be described in terms of the largest droplet volume $V_{\max}$. Then, phase inversion occurs when $V_{\max}$ reaches its maximum available size V_M. By defining V_H the minimum droplet size for $V_{\max}$, we introduce the dimensionless variable:

$$x \equiv \frac{V_{\max} - V_H}{V_M - V_H} \tag{S3}$$

The next step is to model the dynamical behaviour of $x \in [0, 1]$ subject to breakup and

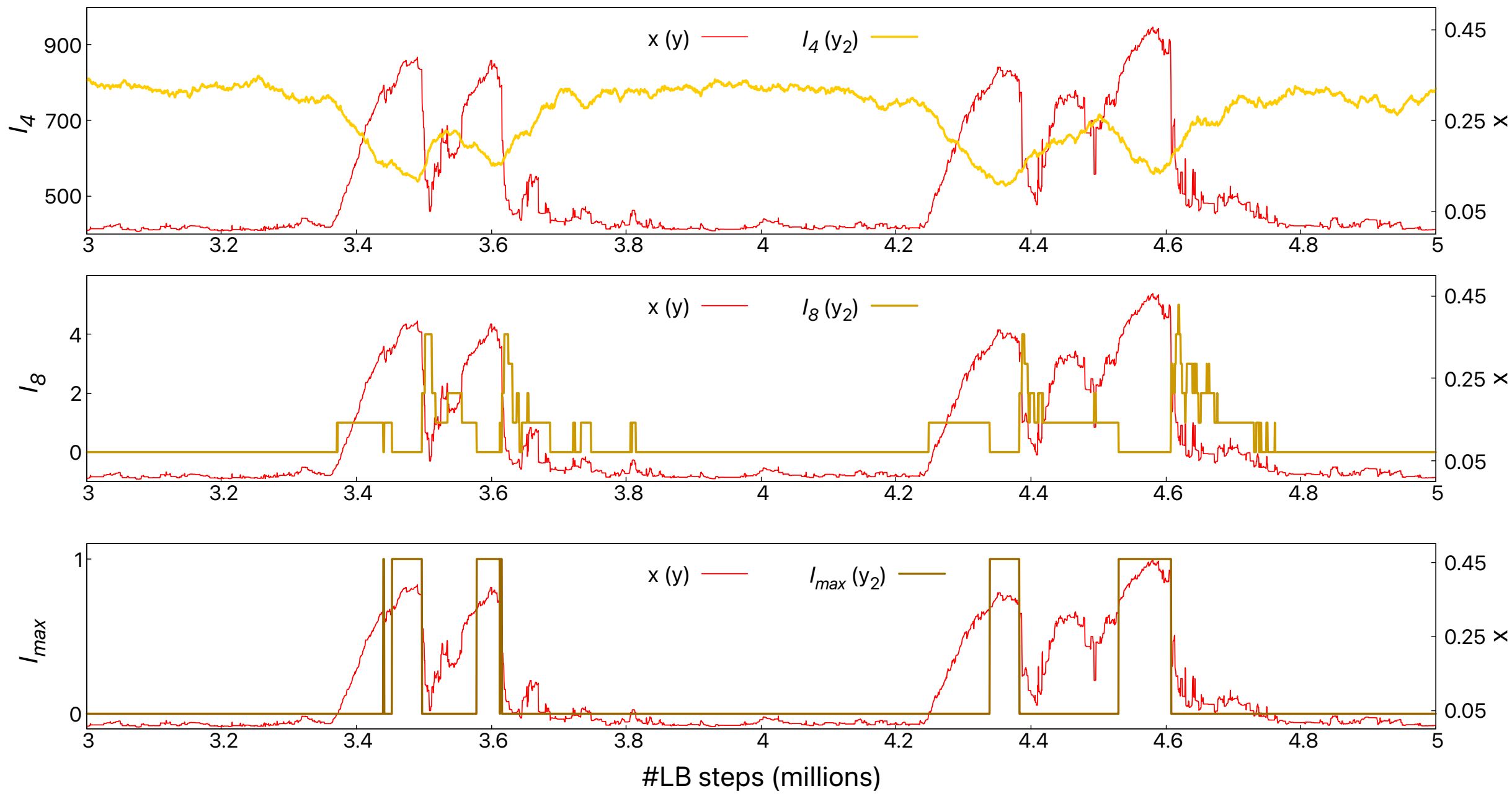

Supplementary Figure 2: A graphical visualization of the temporal evolution of the cumulative distribution $\overline{n}_I(t) = \sum_{i \in I} n_i(t)$ for a few representative size-classes of droplets during a typical run from fully resolved numerical simulations at high volume fraction: from small size ($I_4 = \{i | R_i \leq 4\langle R \rangle\}$), to large size ($I_8 = \{i | 4\langle R \rangle < R_i \leq 8\langle R \rangle\}$) and up to the largest-size ($I_{\max} = \{i | R_i = R_{\max}\}$), where R_i is the radius of the i-th droplet and $\langle R \rangle$ is the mean radius over the ensemble of droplets. The picture displays the population dynamics (y, different shades of yellow) overlapped with the time evolution of the fraction of volume occupied by the largest droplet $x(t)$ (y2, red). It provides evidence of a slower coalescence-driven dynamics coinciding with the formation of a large droplet, followed by a faster breakup-driven dynamics that tends to bring the emulsion back to equilibrium. It shows that during the coalescence-driven phase only a single large droplet is formed in the emulsion that becomes bigger and bigger by including small droplets. Indeed, the number of droplets of the small-size class generally decreases while only one single increasing large droplet is displayed on the plots related to the larger-size classes. On the other hand, the largest droplets breaks up forming multiple large droplets of the same size-class, with this behavior repeating recursively on the newly generated droplets until equilibrium, whether not leading to the formation of a new large droplet region or eventually at a CPI. Source data are provided as a Source Data file.

coalescence. Let ϕ the volume fraction. For $\phi > 50\%$, emulsions are subject to coarsening whose dynamics depends on ϕ, the effects of surfactants (if present) and turbulence. Eventually, for $x = 1$ coalescence process stops. Hereafter, we assume that the above dynamics is described by the equation:

$$\frac{dx}{dt} = \alpha\phi(1 - x) \tag{S4}$$

with $\alpha \geq 0$. Next, let us consider the process of breaking up, which counteracts the dynamics of coalescence. This can be done by writing:

$$\frac{dx}{dt} = (\alpha\phi - fx)(1 - x) \tag{S5}$$

where $f \geq 0$. The above equation is the simplest form of coalescence and breakup dynamics of x. Now, we need to specify the two unknown quantities α and f. From Eq. (S5) we see that, for $\alpha\phi/f < 1$, the fixed point $x_* = \alpha\phi/f$ is stable. Inspection of the inset of Supplementary Figure 2 tells that x is a rather intermittent quantity showing relatively long periods with $x \sim 0$ and bursts of large value of x close to 1. This observation suggests that the ratio α/f should be an intermittent quantity which we need to model. To do that, we assume α to be constant and f to satisfy the stochastic differential equation:

$$df = \left[-rf - \beta f^3 + \frac{1}{4}\frac{d\xi}{df}\right] + \sqrt{\xi(f,x)}dW(t). \tag{S6}$$

A reasonable physical ground for this equation is provided in what follows. The first two terms in the square bracket represent the linear and the non linear decreases of f towards the state $f = 0$ where (for zero noise) the system does not exhibit breakup events. This situation should occurs when x is close to 1. The term $\xi(f,x)$ is the variance of the noise introduced in eq. (S6) to mimic, in a very simple way, the effect of turbulence. Then the last term in the square brackets in eq. (S6) is the Stratonovich term induced by multiplicative noise [5]. For x relatively small, we expect turbulence to destabilize the state $f = 0$. This implies that $\xi(f,x)$ should be at least quadratic in f. Also we expect that this effect disappear for x close to 1. The above discussion suggests the following expression for $\xi(f,x)$:

$$\xi(f,x) = \varepsilon_0 + \varepsilon_1 f^2(1 - x) \tag{S7}$$

Eq. (S6) now reads:

$$df = [-rf - \beta f^3 + \frac{1}{2}\varepsilon_1 f(1 - x)]dt + \sqrt{\varepsilon_0 + \varepsilon_1 f^2(1 - x)}dW(t) \tag{S8}$$

Notice that assuming x constant, the stationary probability distribution $P(f|x)$ is

$$P(f) = \frac{Z}{[\varepsilon_0 + \varepsilon_1 f^2(1-x)]^{1/2+r/(\varepsilon_1(1-x))}} \exp(-N_L) \tag{S9}$$

where Z is a normalization constant and $N_L \sim f^2 + ...$ stands for the contributions of the non linear terms. From Eq. (S9) it is clear that, for $x < 1$, $P(f)$ shows a power law distribution at relatively large f which is the signature of intermittency. Notice that eq.(S7) and eq. (S8) are meaningful for $f \geq 0$, i.e. $\partial_f P(f)|_{f=0} = 0$.

First of all, let us discuss the case $\varepsilon_1 = 0$. In this case, f is spending most of the time near $f = 0$ with a variance $f_s \sim \sqrt{\varepsilon_0/(2r)}$. Then the value of x stabilizes near the value $\alpha\phi/f_s$. Therefore, phase inversion occurs at $\phi_s = f_s/\alpha$. It is simple to check that this is also the critical value of ϕ at which the phase inverted state $x = 1$ is stable for any value of ε_1. Thus ϕ_s should be considered the lower bound at which phase inversion can occur. Let us remark that, for $\varepsilon_1 = 0$, the system exhibits one and only one stable state $\alpha\phi/f_s$ for $\phi \leq \phi_s$ and $x = 1$ for $\phi \geq \phi_s$. In other words, no sharp transition (catastrophic phase inversion) is observed and the system linearly increase the average value of x up to $x = 1$. The situation changes completely for the non-vanishing value of ε_1. From eq. (S8) we can compute the critical value $x = x_s$ at which the linear term in square bracket of eq. (S8) becomes zero. A simple computation gives $x_s = 1 - 2r/\varepsilon_1$. This result can be interpreted by saying that a phase inversion can occur for $\phi \geq \phi_s$ only if x becomes larger than x_s. Notice that when $x \geq x_s$ with $\varepsilon_1 > 0$ the system is characterized by two different time scales: the coalescence time scale $1/(\alpha\phi)$ and the breakup time scale associated to the tail of the probability distribution $P(f)$, i.e. to the characteristic time for f to show an intermittent excursion. The above discussion implies that the transition to the (stable) state $x = 1$ occurs at a critical value $\phi = \phi_c$ with $\phi_c > \phi_s$. Also ϕ_c increases with ε_1. For $\phi < \phi_c$ the system is characterized by the balance of $\alpha\phi$ with $\langle fx \rangle$, where $\langle .. \rangle$ stands for time average, whereas for $\phi > \phi_c$ one has $\alpha\phi > \langle fx \rangle$. Technically this also implies that near the transition there exist two stable states, namely the phase inverted state $x = 1$ and the jammed state $\alpha\phi = \langle fx \rangle$.

To validate the above discussion, we consider the following model parameters: $\alpha = 0.002$, $r = 0.02$, $\varepsilon_0 = 10^{-7}$ and two choices of ε_1 namely $\varepsilon_1 = 0.1$ and $\varepsilon_1 = 0.2$. The value of α is chosen by tuning our model with the LBM simulations. Numerical simulations for $\varepsilon_1 = 0$ gives the estimate $\phi_s = 0.65$. In Supplementary Figure 3 we show the average value of x, denoted by $\langle x \rangle$ a function of ϕ obtained by averaging over 100 noise realizations. In the main

part of the figure we plot $\langle x \rangle$ for ϕ increasing from 0.1 for $\varepsilon_1 = 0$ (circles), 0.1 (triangles) and 0.2 (squares). Upon increasing ε_1 we observe a shift in the value of ϕ_c which increases with ε_1. In the insert we show the quantity $Q \equiv \alpha\phi/\langle fx \rangle$. For $\phi \leq \phi_c$, Q, this ratio is close to 1, i.e. the system shows balance between the coalescence terem $\alpha\phi$ and the fragmentation $\langle fx \rangle$. Close to $\phi = \phi_c$, Q starts to grow while the average value of x increases towards the phase inverted state $x = 1$.

The theoretical discussion and the results shown in Supplementary Figure 3 indicate that the system does exhibit a sharp transition between the jammed state (small $\langle x \rangle$) and a phase inverted state $x = 1$, i.e. a catastrophic phase inversion. Next we discuss how to

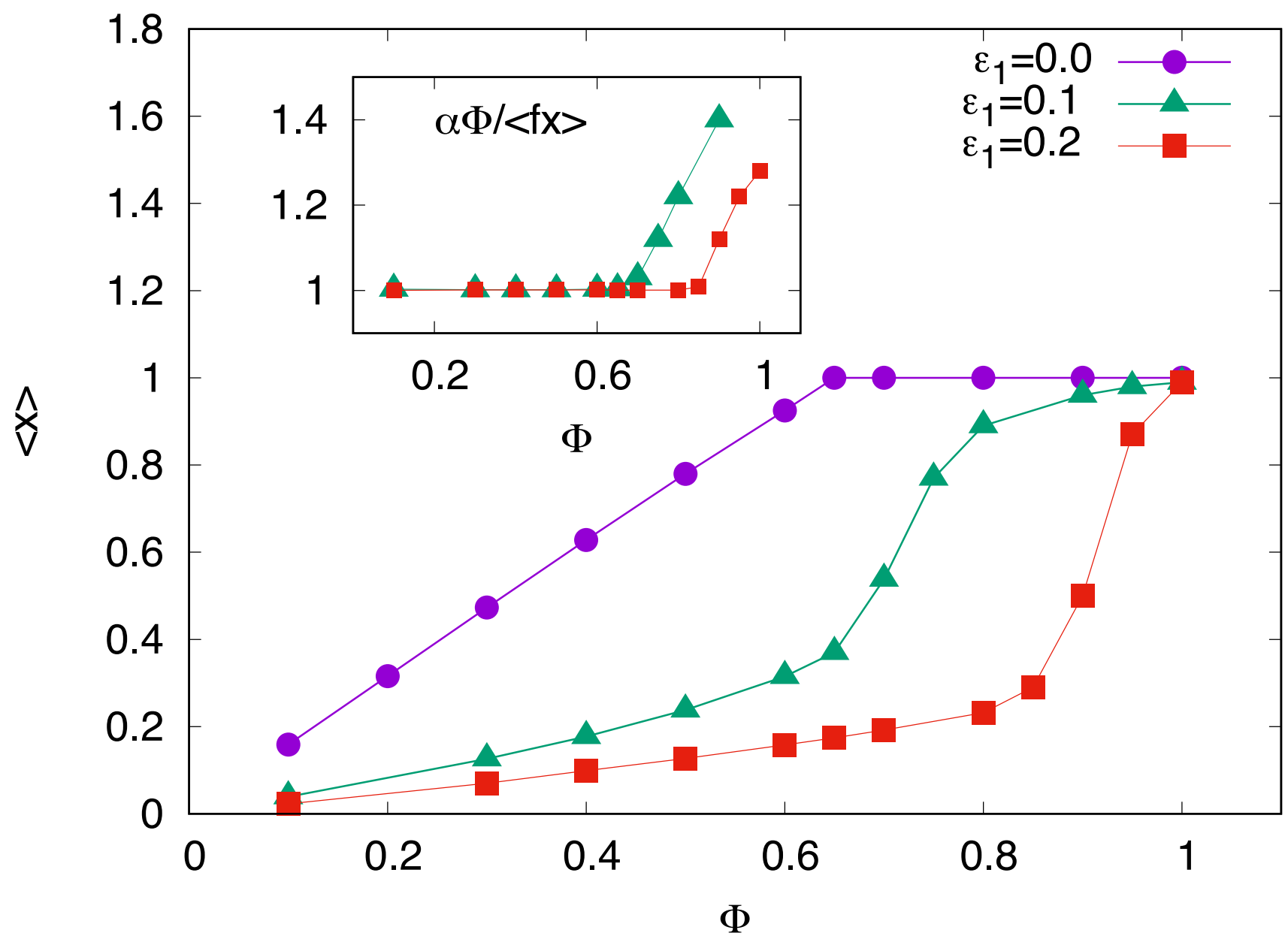

Supplementary Figure 3: Main panel. Behavior of the average value $\langle x \rangle$ for three values of $\varepsilon_1 = 0$ (circles), 0.1 (triangles) and 0.2 (squares) and for fixed $\varepsilon_0 = 10^{-7}$. Notice that for increasing value of ε_1 the transition to $\langle x \rangle = 1$ occurs for larger values of ϕ. Each point in the figure is obtained by averaging in time and over 100 independent realizations. Inset: The ratio of $\alpha\phi/\langle fx \rangle$ as a function of ϕ for $\varepsilon_1 = 0.1, 0.2$ (same symbols as in the main panel). The results clearly show that for small enough ϕ (depending on ε_1) the system shows stationary states for $\langle fx \rangle = \alpha\phi$. Source data are provided as a Source Data file.

compute the parameters $\alpha, \beta, r, \epsilon_0, \epsilon_1$ for Eqs. (S5)-(S8). We would like to emphasise that the dynamics described by equations Eqs. (S5)-(S8) are intentionally oversimplified in order to capture the physical properties of catastrophic phase inversion. A more elaborate model

could obviously be introduced to obtain better and/or more realistic dynamics. However, we do not think that a more elaborate model is useful, given that we already have extremely good numerical simulations, as described in Section B. In order to compute the different parameters in Eqs. (S5)-(S8) firstly we notice that two of the parameters, namely β and ϵ_0, are almost irrelevant. β is introduced to stabilise x at large value (i.e/ to avoid numerical overflow) and even the term βf^3 can be changed in any non linear term which provides stabilisation. The parameter ϵ_0 is introduced to avoid the the state $f = 0$ is an absorbing state , i.e. once the system reaches $f = 0$ remains there for ever. Moreover the value of ϵ_0 is constrained by the condition

$$\phi_s = \frac{1}{\alpha} \left[\frac{\epsilon_0}{2r} \right]^{1/2} \geq 0.5$$

The physical meaning of the above equation is that, assuming that non intermmittent fluctuations characterise the dynamics of the largest droplet (i.e. $\epsilon_1 = 0$ in Eq. (S8)), then the phase inversion may eventually occur for volume fraction ϕ greater than 0.5. Next we need to compute α and r. The value of α can be computed from the equation:

$$\alpha\phi = \langle fx \rangle$$

which holds for relatively small value of ϕ. From the LBM simulations we can compute the r.h.s of the above equation and, knowing ϕ, we can compute α. Again from LBM simulations, we can prepare the system with some given volume fraction ϕ and external forcing. Then, we stop the forcing and we can measure how the rate of fragmentation slows down. The characteristic time of this dynamics provides us the value of $1/r$. The most difficult term to compute from the available data is ϵ_1. Theoretically, the probability distribution of f is characterized by power law decay whose exponent depends on r, ϵ_1 and x see Eq. (S8)). However this is a very cumbersome procedure to employ. It is much simpler to follow the discussion in this section where we show that the phase inversion may occur if x becomes bigger than $1 - 2r/\epsilon_1$. In other words, ϵ_1 can be useed as fitting parameter to fix the value of the critical volume fraction. At any rate since the model depends on many parameters, it is worthwhile to show that it can be recast in a somehow simpler way. Using the quantity f_s, ϕ_s and x_s, upon rescaling the time as $\tilde{t} = rt$, the rate of fragmentation $\tilde{f} = f/f_s$, it is

possible to rewrite the model in the following way:

$$d\tilde{f} = \left[\frac{x_s - x}{1 - x_s}\tilde{f} - \tilde{\beta}\tilde{f}^3\right] d\tilde{t} + \sqrt{2 + 2\tilde{f}^2 \frac{1 - x}{1 - x_s}} dW(\tilde{t}) \tag{S10}$$

$$\frac{dx}{d\tilde{t}} = A\left[\frac{\phi}{\phi_s} - \tilde{f}x\right](1 - x) \tag{S11}$$

where $A = f_s/r$ and $\tilde{\beta} = f_s^2\beta/r$ are dimensionless parameters. The above equations show that there are three independent parameters namely ϕ_s, x_s and A. The latter can be written as $A = \phi_s\alpha/r$ and it is proportional to the ratio of two time scales characterizing the dynamics of the system, i.e. the coalescence and break up processes. Based on the numerical simulations previously discussed (see also [6]) we can assume that coalescence time scale $1/\alpha$ is much longer than the breakup time scale $1/r$ which implies $A \ll 1$ Once the value of A is fixed, the model depends on the two parameter ϕ_s (the lower bound for the phase inversion to occur) and x_c (the value of $V_{\max}$ above which the system may be driven by the noise to a phase inversion). For the numerical results previously discussed, the corresponding value of A in eq. (S11) is $A = 0.08$.

[1] C. Coulaloglou and L. Tavlarides, Drop size distributions and coalescence frequencies of liquid-liquid dispersions in flow vessels, AIChE J. **22**, 289 (1976).

[2] S. Maaß and M. Kraume, Determination of breakage rates using single drop experiments, Chem. Eng. Sci. **70**, 146 (2012).

[3] P. Krapivsky, S. Redner, and E. Ben-Naim, eds., A kinetic view of Statistical Physics (Cambridge University Press, 2010).

[4] N. Brilliantov, P. Krapivsky, A. Bodrova, F. Spahn, H. Hayakawa, V. Stadnichuk, and J. Schmidt, Size distribution of particles in saturn's rings from aggregation and fragmentation, Proc. Natl. Acad. Sci. USA **112**, 9536 (2015).

[5] H. Risken, The Fokker-Planck Equation: methods of solution and applications (Springer International Publishing, 1996).

[6] L. Yi, I. Girotto, F. Toschi, and C. Sun, Divergence of critical fluctuations on approaching catastrophic phase inversion in turbulent emulsions, Phys. Rev. Lett. **133**, 134001 (2024).
