# Supplementary material for: Non-reciprocal coalescence-breakup dynamics in flowing concentrated emulsions
Source: Nat Commun. 2026 Jul 6;17:8339. doi: 10.1038/s41467-026-75077-7 (PMC13473571; doi:10.1038/s41467-026-75077-7)
Supplement: Supplementary file 2 [file 41467_2026_75077_MOESM2_ESM.pdf]

nature portfolio

Peer Review File

Non-reciprocal coalescence-breakup dynamics in flowing concentrated emulsions

Corresponding Author: Professor Federico Toschi

This file contains all reviewer reports in order by version, followed by all author rebuttals in order by version.

Attachments originally included by the reviewers as part of their assessment can be found at the end of this file.

Version 0:

Reviewer comments:

Reviewer #1

(Remarks to the Author)
See my report attached.

Reviewer #2

(Remarks to the Author)
The manuscript examines the rheology and dynamics of concentrated emulsions using a Taylor-Couette turbulent system. Experiments are conducted by progressively adding the dispersed phase to the emulsion to probe the torque response and investigate the phenomenon of phase inversion at large volume fraction. Lattice-Boltzmann simulations are also implemented to improve characterization of droplet interactions, including coalescence and breakup, at various volume fractions. Fluctuations in effective viscosity are compared near the critical volume fraction for phase inversion and a dynamical-system framework based on the largest droplet size is developed to predict the transition. The approach is expected to provide a general statistical framework to describe various phase transitions. Overall, the manuscript presents interesting results about phase inversion between immiscible liquids in Taylor-Couette systems, however data and analysis seem somewhat limited to that particular system and discussion about the competing effect between droplet breakup and coalescence near the transition is not too elaborated. While the authors presents a work a quality using sound analysis and interpretation, the manuscript in its current form appears more appropriate for a specialized journal.

Reviewer #3

(Remarks to the Author)
See my report attached.

Version 1:

Reviewer comments:

Reviewer #3

(Remarks to the Author)
The authors have carefully addressed my previous comments, revising the figures and expanding the text and discussion accordingly. In my assessment, the manuscript now meets the quality standards of the journal, and I therefore recommend it for publication.

Reviewer #5

(Remarks to the Author)
The topic covered in this article is timely and offers some interesting insights into the catastrophic inversion during emulsification process. The authors have partly addressed the reviewers main concerns and the manuscript could suitable

for publisation after considering and including the following items.

1. The main conclusion is that coalescence and breakup of droplets play a major role in the kinetic stability of the system. Now, this main conclusion is a little vague since this is something widely known in the literature and offer no real insight. I strongly recommend the authors to eliminate it or rephrase it from both the abstract and conclusions.

2. The article is presented in the introduction as dealing with the emulsification process while in the conclusion mention that the objective is to study stability after formation "As a specific case,
we focus on stabilized emulsions of immiscible fluids where the aggregation process consists
in droplet coalescence and the breakup of droplets into smaller droplets". Since you are mentioning brakup of droplets, it is clear that you are dealing with emulsion formation. The way the article is written makes very difficult to fully understand the main objective given these contradictory statements and this must be fully revised.

3. One of the reviewers commented that the organization of the article is not adequate and I think that there is room for improvement in the revised version. For example, the introduction is unnecesarily complex and describes aggregating systems at the beginning to slowly moving to the main topic wich is emulsion stability. Also, there is a mistake when saying that "...prone to phase
inversion, namely a microstructural, irreversible, change towards the thermodynamically
favoured configuration, whereby the continuous minority phase becomes disperse". This is simply not true. The final thermodynamic stable configuration is phase separation under the conditions described. Please, rephrase this comment.

4. While I fully understand that the authors are trying to shed light on the process from a physical point of view, there are some important aspects for researchers workin on emulsion stability and formulation that are not present. One is the real size of the droplets. The results showed are normalized by <D_0> but no mention to that value (or I was not able to find it). And the second is the real polydispersity percentage of the emulsions obtained in the experiments. These two variables are key to determine the stability of the system alongside the volume fraction. Depending on the droplet size, Brownian fluctuation, Ostwald ripening, hydrodynamic interaction, and droplet deformability plays diferent roles on the fate of the emulsion. The authors must discuss these aspects and how they consider their results can be framed.

5. The absence of surfactant is fully understandable give the complexity it adds in both experiments and simulations. However, this absence strongly limits the impact of the work and the potential application in more realistic emulsions. The fitting parameters partly contains these pieces of information and has been used in different theories to account for interfacial rheology. The authors can also discuss this aspect and frame their findings accordingly.

Version 2:

Reviewer comments:

Reviewer #5

(Remarks to the Author)
The authors have properly addressed all the questions and modified the article accordingly. The paper is suitable for publication.

Open Access This Peer Review File is licensed under a Creative Commons Attribution 4.0 International License, which permits use, sharing, adaptation, distribution and reproduction in any medium or format, as long as you give appropriate credit to the original author(s) and the source, provide a link to the Creative Commons license, and indicate if changes were made.
In cases where reviewers are anonymous, credit should be given to 'Anonymous Referee' and the source.
The images or other third party material in this Peer Review File are included in the article's Creative Commons license, unless indicated otherwise in a credit line to the material. If material is not included in the article's Creative Commons license and your intended use is not permitted by statutory regulation or exceeds the permitted use, you will need to obtain permission directly from the copyright holder.
To view a copy of this license, visit https://creativecommons.org/licenses/by/4.0/

Dear Editor,

We would like to warmly thank all Referees for the careful reading of our manuscript and the constructive comments that helped us further improve it. We are glad to read that all Referees find the manuscript suitable for publication, and we are confident that we have addressed all their remarks and suggestions.

In the following, we enumerate the comments from the Referees (in black and in quotes) and reply point by point in blue. For convenience, we report in Italic the changes to the manuscript text (new text highlighted in red).

REPLY TO REFEREE 1

"This manuscript investigates the coalescence breakup dynamics of concentrated emulsions approaching catastrophic phase inversion (CPI) through a combined experimental, numerical, and theoretical study. The authors propose a reduced dynamical model where the fraction of volume occupied by the largest droplet acts as an order parameter. They report that the loss of detailed balance between breakup and coalescence leads to non-reciprocal dynamics and critical fluctuations near CPI. The topic is timely and relevant to soft matter and non-equilibrium systems, and the paper is clearly written and well organized. The combination of Taylor-Couette experiments, direct numerical simulations, and a reduced stochastic model is a significant strength. The close correspondence between experimental observations and simulation data convincingly supports the proposed framework. The idea of describing CPI using a non-reciprocal dynamical-system perspective is novel and potentially impactful. However, before the manuscript can be considered for publication, I recommend the following clarifications and improvements:"

Point 1: "The derivation and physical interpretation of the stochastic model (Eqs. 1–2) should be discussed in more detail. How are the parameters α, r, β, ε_0, and ε_1 related to measurable quantities such as coalescence or breakup rates?"

Point 2: "The model assumes that breakup and coalescence can be reduced to two scalar

rates (α, f). This simplification implicitly neglects droplet–droplet hydrodynamic coupling and shape anisotropy near jamming. The authors should justify why this reduction still captures the observed hysteresis and bimodality, or show that including higher order coupling terms does not change the main conclusions.”

Reply to Point 1 & 2: We have fused our answers to these two remarks together, as we deem it convenient for the sake of explanation, being the two, somehow related.
Firstly, the Referee is correct in pointing out that, near the jamming point, there are many droplet-droplet interactions and strong non-isotropic stretching. This is clearly evident in the numerical simulations presented in Section B of the Materials and Methods, which are based on the fully resolved dynamics of the emulsion using the LBE method. The model described in equations (1–2) aims to provide a simplified physical representation of the catastrophic phase inversion observed in experiments and numerical simulations. The basic assumption is that the dynamics of the largest droplet are a suitable order parameter with which to describe the transition. Obviously this assumption must be justified and, precisely for this reason, we show in figure (2) that the size of the largest droplet x can be considered a reasonable "order parameter" to describe the complex underlying dynamics. Next we discuss how to compute the parameters $\alpha, \beta, r, \epsilon_0, \epsilon_1$ for Eqs. (1-2). We would like to emphasise that the dynamics described by equations (1)–(2) are intentionally oversimplified in order to capture the key essence of the physical phenomenology beyond catastrophic phase inversion. A more elaborate model could obviously be introduced to obtain better and/or more realistic dynamics. However, we do not think that a more elaborate model would be useful, given that we already have extremely good numerical simulations, as described in Section B. In order to compute the different parameters in eq. (1-2) we notice firstly that two of the parameters, namely β and ϵ_0, are almost irrelevant. They are introduced to remove possible pathologies but do not play any relevant role in the dynamics. β is introduced to stabilise x at large value (i.e. to limit its growth) and the term βf^3 can be changed in any non linear term which provides stabilisation. The parameter ϵ_0 is introduced to avoid that the state $f = 0$ is an absorbing state, i.e. once the system reaches $f = 0$ remains there for ever. Moreover the value of ϵ_0 is constrained by the condition

$$\phi_s = \frac{1}{\alpha}\left[\frac{\epsilon_0}{2r}\right]^{1/2} \geq 0.5$$

The physical meaning of the above equation is that, assuming that non intermittent fluctuations characterise the dynamics of the largest droplet (i.e. $\epsilon_1 = 0$ in eq. (1-2))), then the phase inversion may eventually occur for volume fraction ϕ greater than 0.5. Next we need to compute α and r. The value of α can be computed from the equation:

$$\alpha\phi = \langle fx \rangle$$

which holds for relatively small value of ϕ. From the LBE simulations we can compute the r.h.s of the above equation and, knowing ϕ, we can compute α. Again from LBE simulations, we can prepare the system with some given volume fraction ϕ and an external forcing. Then, we stop the forcing and we can measure how the rate of fragmentation slows down. The characteristic time of this dynamics provides us with the value of $1/r$. The most difficult term to compute from the available data is ϵ_1. Theoretically, the probability distribution of f is characterized by power law decay whose exponent depends on r, ϵ_1 and X see eq. (14). However this is a very cumbersome procedure to employ. It is much simpler to follow the discussion in section E where we show that the phase inversion may occur if x becomes bigger than $1 - 2r/\epsilon_1$. In other words, ϵ_1 can be used as fitting parameter to fix the value of the critical volume fraction. The above discussion is now reported, in red colour, in section E.

Point 3: "The experimental section should include the type and concentration of surfactant, interfacial tension, and relevant Reynolds/Weber numbers. These are essential for reproducibility and for interpreting the reported critical volume fraction."

Reply to Point 3: We thank the Referee for this suggestion. We have now added these essential details in the revised manuscript. In the section Emulsification at high volume fraction:

In TTC experiments, the Reynolds number can be estimated as $Re = \omega_i r_i d/\nu_0$, where r_i and d are the radius of the inner cylinder and the gap width between the inner and outer cylinders. In our experiments, we have $\phi_c^{(exp)} \approx 92.5\%$ at $Re = 1.0 \times 10^4$.

In the section I A-Experiments:

The interfacial tension between the two immiscible liquids is $\gamma = 5.7 \pm 0.4$ mN/m, which is measured using the pendant drop technique (oil droplet in ethanol-water) on a goniometer instrument (SCA20). No surfactant was intentionally added in the experiments. The closely-packed droplets in the dense emulsion before phase inversion require some kind of disjoining pressure for not fully coalescing. In our experiments, such a stabilizing effect could be sourced from unavoidable surface-active impurities accumulated at the oil–water interfaces, which can effectively suppress droplet coalescence. Possible sources of these surface-active impurities include trace contaminants in the working fluids (i.e., silicone oil and ethanol) and adsorption from the container walls, which has also been discussed in our previous work [1]. We stress that the reproducibility of the results is confirmed by 6 independent experiments. The droplet Weber number is given as $We = \rho {U_{rms}}^2 \langle D \rangle / \gamma$, where ρ is the density of the continuous phase, U_{rms} is the root mean square velocity, $\langle D \rangle$ is the average droplet diameter, and γ is the interfacial tension between the two phases. Based on the experimental measurements, the estimated We is around 2 to 5.

In addition, the relevant experimental parameters for the data shown in the figures are added in the corresponding caption.

Point 4: "The scaling of fluctuations $\sigma \sim (\phi_c - \phi)^{-1/2}$ is intriguing but should be statistically supported. Please provide uncertainty estimates or fit confidence intervals."

Reply to Point 4: We thanks the Referee for this useful remark. In order to give a best fit for the exponent in the relation $\sigma = A\,(\phi_c - \phi)^{\chi}$, we performed a non-linear least square analysis (for $\phi \gtrsim 60\%$), employing the Levenberg-Marquardt algorithm. From the experimental data we got $\chi = -0.50$, with a 90% confidence interval of $\chi = -0.50 \pm 0.04$ (estimated out of the standard error on χ and the associated Student's t-percentile). Analogously, for the theoretical data obtained from the model we got $\chi = -0.50$ with a 90% confidence interval of $\chi = -0.50 \pm 0.03$ and for the numerical data we got $\chi = -0.48$ with a 90% confidence interval of $\chi = -0.48 \pm 0.07$. We have added this information in the caption of Figure 5 as follows:

The dashed line highlights the power law divergence $\sigma \sim (\phi_c - \phi)^\chi$, with $\chi = -1/2$. More precisely, a non-linear least square fitting the function $f(x) = A\, x^\chi$ to the data in the range $\phi \gtrsim 60\%$, using a Levenberg-Marquardt algorithm, provides the following 90% confidence intervals for the exponents: $\chi = -0.50 \pm 0.04$ (experiments), $\chi = -0.48 \pm 0.07$ (numerics) and $\chi = -0.50 \pm 0.03$ (model).

We remark that, of course, a further possible source of error comes from the uncertainty on the value of ϕ_c. Since its determination is highly accurate in the experiments and in the model, we work out the case of the LBM simulations, where we can locate ϕ_c in the range $(77.5 \pm 1)\%$. Performing the fit for selected values in that interval, we get a set of best estimates for χ (with associated standard error σ_χ), gathered in the following table

ϕ_c	χ	σ_χ
76.5%	0.53	0.03
77.0%	0.46	0.04
77.5%	0.48	0.04
78.0%	0.54	0.04
78.5%	0.55	0.04

We see that there is indeed some dependence of the fit on the chosen value of the critical volume fraction, but, within errors, all seem to be consistent with the $-1/2$ scaling. In the revised manuscript we have rephrased, with more cautious words, the paragraph that mentions the scaling in the main text as follows:

In all cases, we observe a divergent behaviour of σ as a function of the distance from the CPI transition point ϕ_c which appears consistent, within statistical errors, with the power law $\sigma \sim (\phi_c - \phi)^{-1/2}$.

Point 5: "The term "non-reciprocal phase transition" could be explained more clearly. A short comparison with recent literature on non-Hermitian or active matter transitions would help situate the work."

Reply to Point 5: We fully agree with the Referee that the extensive recent developments

in the field of non-reciprocal phase transition cannot be easily summarized here for those who are not familiar with the subject. Our goals was rather to indicate a direction on how to interpret the dynamic of this system and refer the interested reader to the relevant literature on the subject.
We have now expanded the section where we mention the connection between the dynamics of our system and the dynamics of some induced rotating phase observed, e.g. in active matter systems.

Our study highlights a certain analogy between CPI and non-reciprocal phase transitions [2], out-of-equilibrium transitions fundamentally distinct from equilibrium ones whose dynamics is governed by free energy minimization. In active matter, non-reciprocal systems exhibit time-dependent dynamical phases that induce chiral rotating phases and active time crystals absent in reciprocal systems [3]. These transitions unify non-Hermitian topology and active matter critical phenomena, describing collective behavior in systems that escape energy optimization principles. In our system, the asymmetric role of breakup and coalescence in the droplet population dynamics breaks microscopic detailed balance and, at the mathematical level, induces non-Hermitianity in the Jacobian of the deterministic limit ($W_t = 0$) of system (1)-(2) [2, 4].

Point 6: "The introduction could be condensed by removing background repetition and focusing more sharply on how this study advances understanding beyond classical population balance or catastrophe theory descriptions."

Reply to Point 6: We thank the Referee for this remark. We have reshaped the introduction of the revised version taking it into account. Specifically we have removed the following sentences (struck through in the revised text):

Emulsions are soft materials, widespread in biological and industrial processes [5], consisting of a liquid-liquid dispersion, stabilized by the presence of surfactants (see Fig. 1).

The phase inversion may occur in a smooth transitional way, typically when it is trig-

gered by changes in various parameters (such as temperature, pH, chemical affinity), that affect the distribution of surfactant over the phases [6, 7].

Catastrophe theory is essentially a bifurcation theory for the dynamics of a behaviour variable (related in some sense to the emulsion morphology, in the case of phase inversion), occurring in the space of control parameters which define the optimization function (a generalized effective free energy) [6].

Point 7: “The DNS comparison relies on a diffuse interface approach, yet the interface width correction is mentioned only qualitatively. Quantify how sensitive the predicted critical volume fraction (ϕ_c) is to interface resolution and to the choice of surface tension parameters.”

Reply to Point 7: The Referee’s remark is correct. Indeed, the diffuse nature of the interface should be taken into account, if one aims at quantitative comparisons with experiments, when it come to the precise determination of the critical volume fraction. Although the main intent of our work is to show the universal behaviour of global observables at approaching the critical point (rather than the prediction of the critical point itself), we do agree that this aspect deserves attention. That is why we dedicated a section to it (section C of Materials and Methods: “The volume fraction corrections in numerical simulations”). Following the Referee’s suggestion, we add some more details to the discussion. First of all, determining the volume fraction in diffuse interface numerical approaches requires the definition of a threshold. As customary, we set it to the mean between the extremal density values of each of the two fluids, i.e. we assign the label, e.g., "oil" to each lattice point $\mathbf{x}$ such that $\rho_O(\mathbf{x}) \geq \overline{\rho}_O \equiv (\rho_O^{(\max)} + \rho_O^{(\max)})/2$. The volume fraction of oil is then the number of points fulfilling this condition over the whole volume; in formulas $\phi_O = \frac{1}{V}\sum_{\mathbf{x}} \Theta(\rho_O(\mathbf{x}) - \overline{\rho}_O)$. This prescription effectively defines a "sharp" interface in the middle of the "actual", diffuse interface. However, especially when droplets are closely packed, the finite width, δ, is relevant in the interaction between neighbouring interfaces. To account for it, we attribute to the droplets (assumed spherical, for simplicity) an "effective" radius $R + \delta/2$. This introduces a correction to the volume fraction, that can be estimated as $\tilde{\phi}_O = \frac{4\pi}{3V} N_D (R + \frac{\delta}{2})^3 = \frac{4\pi}{3V} N_D R^3 (1 + \frac{\delta}{2R})^3 = \phi_O (1 + \frac{\delta}{2R})^3$, where N_D is the number of

droplets. Introducing the numerical values, in lattice spacing, for the interface width, $\delta \approx 3$, and the mean droplet radius, $R \approx 30$, we get, for the corrected critical volume fraction, $\tilde{\phi}_c^{(\mathrm{sim})} \approx 90\%$. We have fully rewritten the section C of Materials and Methods providing a more quantitative discussion as outlined in this reply.

Concerning the second question, the critical volume fraction is indeed expected to depend also on the surface tension, or, more precisely, on the relative amplitude of the surface tension and the hydrodynamic stresses induced on the interfaces by the fluid flow, which can be quantified in terms of dimensionless groups such as the Weber number (ϕ_c grows with decreasing We). In the original version of the manuscript we actually commented on this feature, in the section on "Emulsification at high volume fraction" (page 6), where we wrote "*It must be remarked that the actual value of ϕ_c depends on the flow conditions and on the physico-chemical properties of the interface (encoded in the dimensionless Reynolds, Re, and Weber, We, numbers)*". In the revised version we have rewritten the whole paragraph, providing more quantitative details about both experimental and numerical data, as follows:

It must be remarked that the actual value of ϕ_c depends on the flow conditions and on the physico-chemical properties of the interface (encoded in the dimensionless Reynolds, $Re = U_{rms}L/\nu$, and Weber, $We = \rho U_{rms}^2 \langle D \rangle / \gamma$, numbers, where U_{rms} is the root mean square velocity, L is a characteristic large scale length, $\langle D \rangle$ is the mean droplet diameter, ν and γ are the kinematic viscosity and surface tension, respectively) [6–9]. Moreover, due to the diffuse interface nature of the numerical model, if a quantitative matching between experiments and simulations is sought for, a corrected effective volume fraction, taking into account the finite interface width, should be considered, as discussed in the Materials and Methods sections. Therefore, in what follows we will always refer to $\phi^{(exp)}$ and $\phi^{(sim)}$ for the volume fractions in experiments and DNS, respectively, whenever actual numerical values are involved. In TC experiments, the Reynolds number can be estimated as $Re = \omega_i r_i d/\nu_0$, where r_i and d are the radius of the inner cylinder and the gap width between the inner and outer cylinders. In our experiments, we have $\phi_c^{(exp)} \approx 92.5\%$ at $Re = 1.0 \times 10^4$ [...] The value of the critical volume fraction in the simulations can be, then, estimated as $\phi_c^{(sim)} \approx 77.5\%$

REPLY TO REFEREE 2

"The manuscript examines the rheology and dynamics of concentrated emulsions using a Taylor-Couette turbulent system. Experiments are conducted by progressively adding the dispersed phase to the emulsion to probe the torque response and investigate the phenomenon of phase inversion at large volume fraction. Lattice Boltzmann simulations are also implemented to improve characterization of droplet interactions, including coalescence and breakup, at various volume fractions. Fluctuations in effective viscosity are compared near the critical volume fraction for phase inversion and a dynamical system framework based on the largest droplet size is developed to predict the transition. The approach is expected to provide a general statistical framework to describe various phase transitions. Overall, the manuscript presents interesting results about phase inversion between immiscible liquids in Taylor-Couette systems, however data and analysis seem somewhat limited to that particular system and discussion about the competing effect between droplet breakup and coalescence near the transition is not too elaborated. While the authors presents a work a quality using sound analysis and interpretation, the manuscript in its current form appears more appropriate for a specialized journal."

First of all we would like to thank the Referee for the positive comments regarding the quality of our work. The reviewer comments on the following two points: 1) the *data and analysis seem somewhat limited to that particular system.* 2) the *discussion about the competing effects of droplet breakup and coalescence near the transition is not too elaborated.*

Reply to Point 1: We start by discussing item 1). In our paper we are interested to understand the physics of Catastrophic Phase Inversion for an emulsion subject to external *large scale* forcing. For this reason, we focus on two general cases: a) Taylor-Couette turbulent flows; b) homogeneous isotropic turbulence. Note that in the case of Taylor-Couette flows, the system is characterised by large-scale shear flows and two boundary layers. Therefore, the two turbulent flows (a) and (b) are rather different. In fact, one could say that most turbulent flows in experiments and/or numerical simulations resemble one of these two cases. For example, turbulent channel flows and flows produced by the so-called

"French washing machine" are physically similar to turbulence observed in Taylor-Couette flows, whereas turbulence produced in a wind tunnel, flow downstream of a grid is similar to homogeneous isotropic turbulence. Thus, instead of focusing on a single, particular system, we are studying two very different and somehow opposite situations. To clarify this point, we have included the following paragraph on this in the introduction to the paper, making it clear that the two flows have rather different physical turbulence mechanisms:

Two prototypical cases are considered: turbulent Taylor-Couette flows (experiments) and homogeneous isotropic turbulence (DNS); these two types of flows are chosen precisely in view of their relevant differences (e.g., presence/absence of large scale shear flows and boundary layers), thus supporting the generality of our findings.

Reply to Point 2: Let us now consider item 2). In all our paper we discuss the coalescence and breakup mechanisms in the systems. Figures 2, 3, 4, 6, 9 and 10 clearly show the dynamics of coalescence and breakup, as well as their effect on the droplet populations. In particular, given the observed behaviour of the coalescence and breakup mechanisms as the volume fraction ϕ increases, we propose that a suitable order parameter with which to study phase inversion is the size, x, of the largest droplet. This is an important point because it allows us to highlight the non-reciprocal nature of the phase transition (see Figure 4b), which our simplified model (see Equations 1 and 2) captures. Moreover, we want to stress, as shown in figures 4 and 10, that the mechanism of phase transition is characterized by rather intermittent fluctuations of x, which implies different times scales in the competition between coalescence and breakup mechanism (an observation that we use when developing the model). At this stage, it is difficult to understand how a more detailed description can be achieved. We also remark that quantitative measurements of the rate of breakup and coalescence at approaching the critical volume fraction, in the same computational system, have been already published by some of the Authors elsewhere (I. Girotto et al, *J. Fluid Mech.* **986**, A33 (2024)). In the revised manuscript we have added the following paragraph in order to highlight these aspects related to the breakup and coalescence dynamics:

With the increase of the volume fraction ϕ, the global rates of both coalescence and breakup

show a steep growth, consistent with a diverging power-law function (as reported in [10]). The instantaneous imbalance of the two rates eventually leads to the emulsion destabilisation and CPI. We posit that, as we approach CPI, the "kinetic"-level description provided by the Smoluchowski approach might be further simplified, focusing only on the dynamics of the volume, $M(t)$, of the largest droplet.

REPLY TO REFEREE 3

"The manuscript investigates catastrophic phase inversion (CPI) in dense stabilized emulsions through a combination of Taylor–Couette experiments, direct numerical simulations, and a reduced dynamical systems model. The authors argue that close to the inversion threshold, the emulsion dynamics can be captured by a single coarse-grained variable, specifically the size of the largest droplet, which acts as a dynamical order parameter. They report a transition from Gaussian to bimodal fluctuations in both effective viscosity and droplet size statistics, interpreted as signatures of a non-reciprocal dynamical phase transition governed by competing coalescence and breakup processes. Strengths: The topic is timely, and understanding CPI is of clear relevance for soft matter physics, rheology, emulsion formulation, and consequently many industrial applications such as food processing or care products. The combination of experiments, advanced simulations, and minimal modeling is appealing. Both the scientific topic and methods are potentially a good fit for the broad audience of Nature Communications. Specific Issues, Weaknesses, and Recommendations: However, in its current form, the manuscript falls short in several aspects, making it hard to recommend for publication: it suffers mostly from presentation and organizational issues, as well as from incomplete or unclear explanations: many claims are made without sufficient demonstration or (quantitative) support. Major revisions are needed. My comments and suggestions are listed below, including both major points and more specific remarks following the chronological order of the manuscript."

Point 1: "One of my major points concerns the structure of the article: the authors present experiments and then attempt to interpret them with advanced simulations. As it stands, experimental results are introduced with mini- mal detail in the main text (relying on the Methods section), which leads to two problems:
(i) the experimental results are difficult to follow and understand
(ii) the connection to the simulations, which are meant to interpret underlying mechanisms, is unclear.
(a) I suggest merging Figures 1 and 2 to provide a single integrated view of the experimental findings. This merged figure should include the experimental setup, temporal evolution of key metrics (largest droplet size, rms velocity, effective viscosity, etc.), and representative

snapshots showing morphological changes over time. Including the experimental sketch from Fig. 8(a) would also help clarify the setup. This integration will make the paragraph starting around line 78 more readable.
(b) Fig. 1(b): The emulsion morphology change is not visually clear. Adjust contrast or add annotations to make droplet size and distribution changes more obvious."

Reply to Point 1: We thank the Referee for this insightful comment. For (i), we have now presented a summarized experimental description in the main text (Section-Emulsification at high volume fraction):

In experiments, the oil-water emulsion was produced in a Taylor-Couette flow system confined between two coaxial cylinders, where the outer cylinder is stationary while the inner cylinder rotates at a constant angular velocity to generate a turbulent emulsion (see Section I A). We gradually injected the dispersed phase into the system at a constant volume flow rate to slowly increase its volume fraction ϕ *(a quasi-static process, see Section I A for details), and the instantaneous dispersed-phase volume fraction is given as a function of the injected liquid volume* V_{in}*, i.e.,* $\phi = 1 - e^{-V_{in}/V_{tol}}$*, where* V_{tol} *is the total volume of liquid in the system.*

For (ii), we have strengthened the connection to the simulations in the revised manuscript.
(a) We really agree that a merged figure can provide an integrated view of the experimental findings. Accordingly, we have created a new Fig. 1, which now combines the experimental setup (previously shown in Fig. 8(a)) with the evolution of the effective viscosity. However, merging all panels from the former Figs. 1 and 2 into a single figure may result in an excessively large and potentially less readable figure. To maintain clarity, panels (e,f) from the original Fig. 1 have been moved to the current Fig. 2, which now focuses on integrating the numerical simulation results.
(b) The snapshots of the emulsion morphology in the former Fig. 1(b) (now Fig.1(d)) have been adjusted for a visually clear demonstration.

Point 2: "Lines 35-40: The discussion of external forces driving aggregation and fragmentation is useful, but I am surprised there is no mention of droplet formation (e.g., sprays) or

general particle aggregation, with reference to canonical aggregation theory (Smoluchowski). Including these references would strengthen the context."

Reply to Point 2: We agree with the Referee that mentioning droplet formation phenomena, such those occurring in sprays or liquid jets atomization, is worth and relevant. We believe, though, that, as compared to the phenomena listed in lines 35-40, whose common feature is the transition to a large scale aggregate, atomization is kind of the opposite process. We have therefore made reference to it in the revised version when the population balance equations are first introduced (in the section about 'Droplet population dynamics at approaching CPI'), adding the following sentence:

From the perspective of emulsion morphology, this extremely complex spatiotemporal dynamics can be reduced to a set of balance equations of Smoluchowski type for droplet populations (probability distributions), $n_i(t)$ on the discrete space of size classes [11–14] (see also I D for more details), as proven successful also for phenomena such as spray formation and, more in general, atomization of liquid jets and sheets [15–17].

Point 3: "Line 88: While further details are provided in the Methods section, the main text must provide essential experimental information. In particular, the relation between injected volume and volume fraction (Eq. 3) should be clarified: it is non-trivial and important for reproducibility."

Reply to Point 3: We thanks the Referee for this comment. We have now provided essential experimental information in the main text (see reply to point 1).

Point 4: "Line 93: The statement that "the population is relatively static" should be directly demonstrated in the figures. Merging Fig. 1 and 2 would help clarify how both experiments and simulations evolve over time."

Reply to Point 4: We thanks the Referee for this remark. We agree that it does not seem supported by data as it stands; however, we would like to stress that at that point,

we did not refer to our study, but we were rather reporting what it is well known from emulsification and atomization/fragmentation (see, e.g. [16]). What we meant to affirm is that, in dilute/semi-dilute conditions it is possible to attain a stable stationary droplet size distribution. At increasing the volume fraction, such steady state becomes unstable, for the emulsion is prone to the catastrophic phase inversion. We show that these considerations apply also to our study in Figure 2, plotting the number of droplets. In order to clarify this aspect, we have rephrased the paragraph as follows:

Under these not too high concentration conditions, it is possible to attain a stable, weakly fluctuating, stationary droplet size distribution [16]; we call it, then, a "static" population regime.

Point 5: "Page 5, Fig. 1(a): This figure could be made more quantitative. Many empirical or theoretical models describe the dependence of effective viscosity on oil concentration (dilute, semi-dilute, concentrated regimes). A comparison to such models would be informative; authors knows a priori (figure 9 in the supplementary materials what is the droplet size dependence with the oil concentration). Additionally, the manuscript should discuss reproducibility: is only one experiment shown? Since conclusions rely on statistical distributions, reproducibility is essential."

Reply to Point 5: We thank the Referee for the suggestion on the quantitative comparison of the effective viscosity to the existing models. We have now compared our viscosity data (Fig. 1 in the main manuscript) to both the Taylor relative viscosity $\eta_r = 1 + 2.5\phi(\lambda + 2/5)/(\lambda + 1)$ [18] (λ viscosity ratio (dispersed/continuous)) and the modified Krieger-Dougherty viscosity $\eta_r \left(\frac{2\eta_r+5\lambda}{2+5\lambda}\right)^{3/2} = (1 - \phi/\phi_m)^{-2.5\,\phi_m}$, where $\eta_r = \nu_e/\nu_0$ and the maximum packing fraction $\phi_m = \phi_c$ [19, 20].

The following discussion has been added to the revised manuscript:

We have compared our viscosity data with the Taylor model for dilute emulsions without droplet interactions [18] and the modified Krieger–Dougherty (K-D) relation for concentrated emulsions [19, 20] (Fig. 1). The Taylor model only describes the viscosity evolution

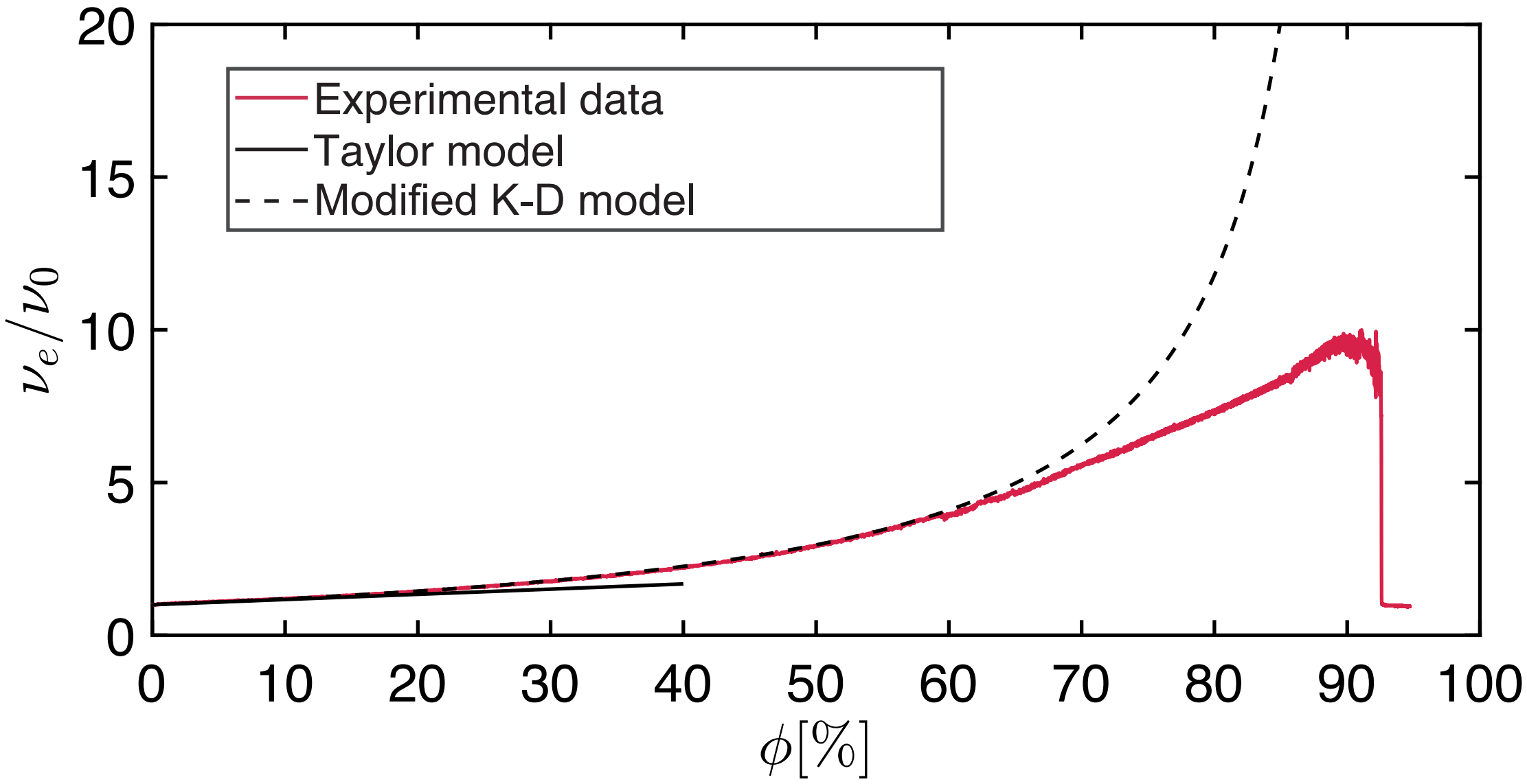

FIG. 1: The quantitative comparison of the effective viscosity as measured in experiments against phenomenological models. The solid black line shows the Taylor relative viscosity $\eta_r = 1 + 2.5\phi(\lambda + 2/5)/(\lambda + 1)$ [18] (λ viscosity ratio (dispersed/continuous)), and the dashed black curve denotes the modified Krieger-Dougherty viscosity $\eta_r \left(\frac{2\eta_r+5\lambda}{2+5\lambda}\right)^{3/2} = (1 - \phi/\phi_m)^{-2.5\,\phi_m}$, where $\eta_r = \nu_e/\nu_0$ and the maximum packing fraction $\phi_m = \phi_c$ [19, 20]. Here $Re = 5.2 \times 10^3$.

at low volume fractions ($\phi \lesssim 10\%$). Interestingly, using the maximum packing fraction $\phi_m = \phi_c$, the modified K-D model captures the experimental data very well until $\phi \approx 65\%$ (static population regime), after which the model overestimates the viscosity in the dynamic population regime where the droplet breakup and coalescence plays an important role. In addition, it should be noted that our effective viscosity is measured in a turbulent emulsion flow, which differs from the laminar flow conditions under which these classical shear-viscosity models are formulated.

We thank the Referee for emphasizing reproducibility. We performed two kinds of experiments: 1) continuous experiments (Fig. 1 in the manuscript), where we continuously and slowly (quasi-statically) vary ϕ, and 2) step-by-step experiments (Fig. 5 and Fig. 6 in the manuscript), where we measured the torque at a fixed volume fraction ϕ for a duration Δt at each step, while ϕ is increased stepwise until the phase inversion occurs. Both ap-

proaches yield reproducible results and lead to reliable conclusions. We have clarified this point in Section I A-Experiments in the revised manuscript.

We clarify that both the continuous experiments and step-by-step experiments yield reproducible results and lead to reliable conclusions. In particular, the step-by-step measurements supported include 6 independent experiments, including 5 experiments with a step duration of $\Delta t = 20$ *min and 1 experiment with a step duration of* $\Delta t = 60$ *min. All repeated experiments yield consistent results and support the conclusions of the manuscript. The reproducibility is explicitly reflected in Fig. 5, where the fluctuations near the phase inversion are shown with error bars that quantify the variability across repeated experiments.*

Point 6: "Line 98: "Through time-resolved global torque"—this must be demonstrated in the main text. Relevant information from the Methods/Supplementary could be summarized here."

Reply to Point 6: We thank the Referee for this comment. The detailed calculation

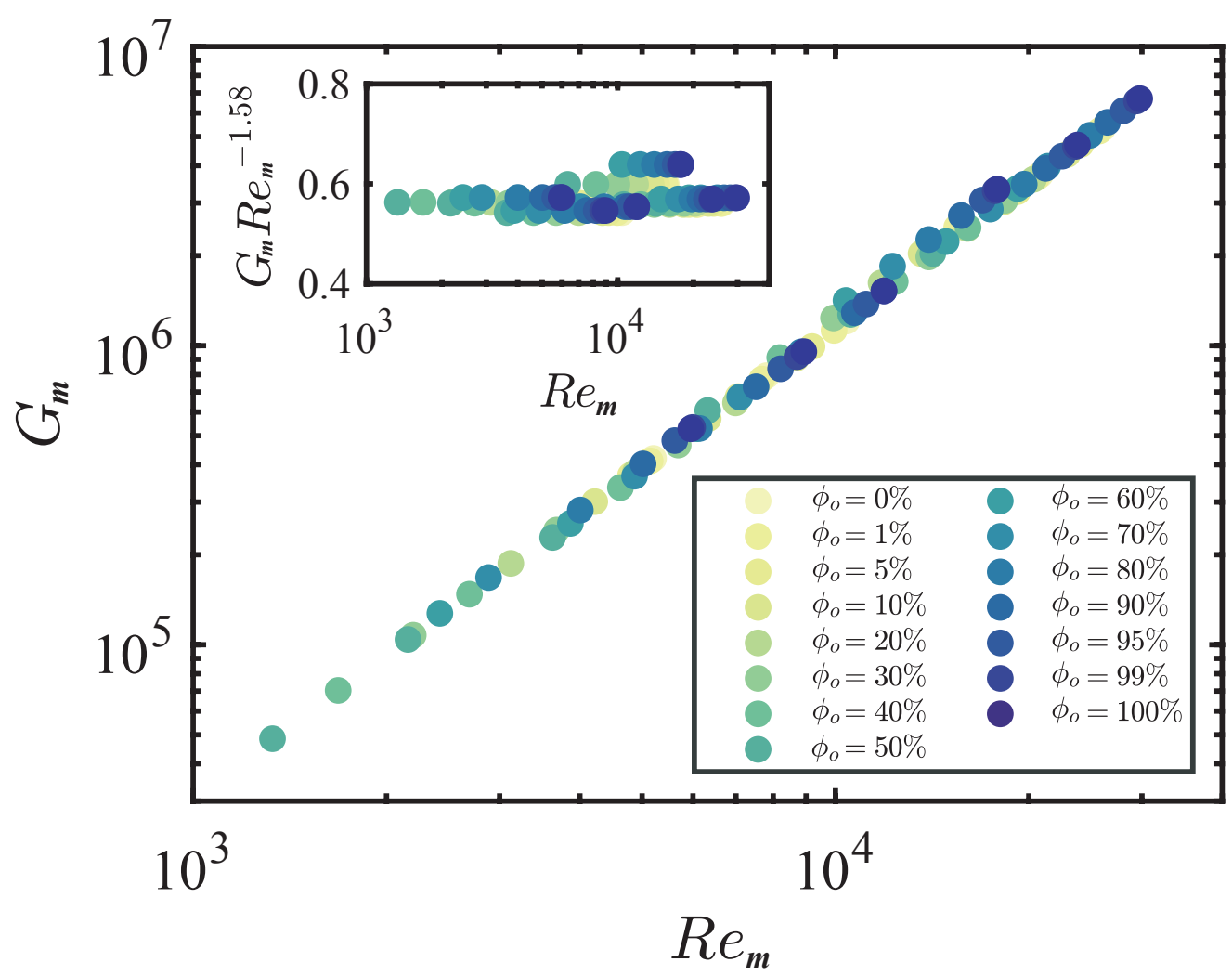

FIG. 2: The dependence between the modified dimensionless torque G_m and the modified Reynolds number Re_m, using the effective viscosity ν_e. All these datasets at the various oil volume fractions ϕ collapse in a master curve. The inset denotes a compensated plot with $G_m \sim {Re_m}^{1.58}$.

of effective viscosity is provided in Section I A, Experiments. We have now demonstrated this essential point in the main text by summarizing the relevant information from the Methods/Supplementary. In Section-Emulsification at high volume fraction:

For a given volume fraction ϕ, the effective viscosity of the emulsion system ν_e can be calculated as $\nu_e/\nu_0 = (T/T_0)^{2.4}$, where T is the corresponding global torque T required to maintain a constant angular velocity (ω_i) of the inner cylinder in our TC system, ν_0 and T_0 are the viscosity of the continuous phase ($\phi = 0\%$) and the torque of the system with $\phi = 0\%$ at the same rotational angular velocity, respectively. This method is based on the assumption that, for various ϕ and Re, the momentum transport between the inner and outer cylinders in the TC system can be described by the same mechanism, of which the details can be found in Section I A.

In Section I A Experiments.:

Here we define the modified dimensionless torque as $G_m = T/(2\pi L\rho{\nu_e}^2)$ and modified Reynolds number as $Re_m = \omega_i r_i d/\nu_e$. For the pure ethanol–water mixture ($\phi = 0\%$) with a known viscosity $\nu_e = \nu_0$, we observe a scaling law $G_m \sim Re_m^{1.58}$ (see Fig. 2). This gives $G_m = K{Re_m}^{1.58}$, where K is a constant prefactor. With the definitions of G_m and Re_m, we obtain a dependence of the torque T_0 on the viscosity ν_0 as $T_0 = AK{\nu_0}^{0.42}$ ($\phi = 0\%$), where $A = 2\pi L\rho/(\omega_i r d)^{0.42}$. This relation is expected to be valid for emulsions with various oil volume fractions ϕ and Re [9, 21]. Thus, for a given ϕ and Re, the effective viscosity of the emulsion system ν_e can be calculated with the corresponding global torque T as

$$\frac{\nu_\mathrm{e}}{\nu_0} = \left(\frac{T}{T_0}\right)^{2.4}. \tag{1}$$

We calculate G_m and Re_m using the effective viscosity obtained for each case, and find that all datasets at various ϕ collapse onto a master curve (see Fig. 2).

Point 7: "Lines 104-107: Statements regarding "significant droplet deformation" and "droplet size" must be visually supported."

Reply to Point 7: We thank the Referee for this remark. We have adjusted the experimental snapshots in Figure 1 to make the droplet deformation and droplet size distribution more apparent. To ensure a more accurate description, we have removed the term "significant" and now refer simply to droplet deformation and droplet size in the revised manuscript.

Point 8: "Line 112: Droplet "lifetime" is introduced abruptly without prior definition. This paragraph is unclear and should be rewritten to convey a structured message."

Reply to Point 8: We are sorry for the lack of clarity. In the revised version, we have rephrased the paragraph as follows:

Moreover, if we define the characteristic droplet 'lifetime' as the mean interval of time between two consecutive breakup/coalescence events involving the same droplet, which can be estimated as $t_D = \langle N \rangle / \langle \beta \rangle$ *(where* $\langle N \rangle$ *is the mean number of droplet and* $\langle \beta \rangle$ *the mean breakup rate), it turns out from our numerical simulations (see Fig. 3 and Table 1 in [10]), interestingly, that* $\phi \approx 65\%$ *corresponds, approximately, to the volume fraction at which* t_D *becomes of the order of the large eddy turnover time, suggesting that the hydrodynamic flows and the interfacial dynamics start to get tightly coupled.*

Point 9: "Lines 116, 124-125: Reynolds and Weber numbers should be defined when first introduced."

Reply to Point 9: We have added the requested definitions with the following sentence:

(encoded in the dimensionless Reynolds, $Re = U_{rms} L/\nu$*, and Weber,* $We = \rho U_{rms}^2 \langle D \rangle / \gamma$*, numbers, where* U_{rms} *is the root mean square velocity,* L *is a characteristic large scale length,* $\langle D \rangle$ *is the mean droplet diameter,* ν *and* γ *are the kinematic viscosity and surface tension, respectively)*

Point 10: "Fig. 2: Consider adding a vertical axis indicating increasing oil concentration. Define the right-axis units (speed) and clarify whether the rms speed refers to the largest

droplet, all droplets, or some other measure (centroid, center-of-mass?). The rationale for focusing on the largest droplet should also be explicitly stated in the text."

Reply to Point 10: Fig. 2 has been changed accordingly (not touched the text yet).

Point 11: "Line 168: Results in Figures 2 and 4 are compared verbally but not directly or plotted together. Consider plotting them together for clarity."

Reply to Point 11: We think that this comment was triggered by the sentence: "Figures 2 and 4(a) tell that the dynamics of $x(t)$ displays bursting events where x reaches large values ($x \sim 0.5$), spaced out by relatively long periods where it remains small ($x \sim 0$)"; but we did not mean to compare the two figures. In fact, the data shown in Fig. 4(a) come from the same simulation of the data in Fig. 2, middle panel, plotted on an extended time interval. We deem, therefore, redundant to plot figures 2 and 4 together, as well as mentioning Figure 2 in the above sentence. We thank, then, the Referee for spotting this redundancy and we removed the reference to Figure 2 from the phrase, which now reads as follows:

Figure 4(a) tells that the dynamics of $x(t)$ displays bursting events where x reaches large values ($x \sim 0.5$), spaced out by relatively long periods where it remains small ($x \sim 0$).

Point 12: "Line 175: "CPI is transition is appealing" – please rephrase for clarity."

Reply to Point 12: We would like to draw the Referee's attention to the full sentence, saying "The idea of identifying x as the relevant order parameter for the CPI transition is appealing, but deserves a more constructive justification", i.e. it is the 'idea' which is appealing, not the CPI transition. Why we deem it 'appealing' deserves certainly some justification, which is given in the follow up of the paragraph.

Point 13: "Figures 3 and 4: Consider merging these figures for clarity. Explicitly state whether time is adimensionalized. Provide the time step between points in Fig. 4."

Reply to Point 13: We thank the Referee for this suggestion. However, we respectfully disagree that clarity would benefit from merging the two figures. Figure 3 is a qualitative one: it is meant as a visual aid to explain the conceptual path leading to the proposed *model* for $x(t)$. Figure 4, instead, aims at describing quantitatively such dynamics, measured form the *direct numerical simulations*, showing a time signal of $x(t)$ and the relative phase portrait. We hope that the Referee will understand our will to keep, then, the two figures separated. Concerning the time, it is indeed adimensionalized, by T_L (as explicitly written in the x-axis label of Figure 4(a) and in the y-axis label of Figure 4(b)), defined in the caption of Figure 4. We have remarked the adimensionalisation in the revised version rewriting the caption as:

(a) The signal of $x(t)$ as a function of time, given in units of the characteristic large scale time $T_L = L/U_{rms}$, from a simulation at $\phi^{(sim)} \approx 77\%$;

We provide now also the information (in the caption) about the time interval between points in Fig. 4(b), which is of 1600 lattice Boltzmann integration steps.

Point 14: "Lines 217-220: The discussion of characteristic times (Wiener processes?) is unclear. Provide context and connect explicitly to the dynamics shown in figures. Can the authors comment on deviations at large $\phi - \phi_c$?"

Reply to Point 14: Scaling in critical phenomena, even at equilibrium, are only to be expected *near* the critical point (ϕ_c in this case). We believe, therefore, that deviations at large $\phi_c - \phi$ are not surprising, particularly when it comes to the model, which is somehow intrinsically designed to work in the 'dynamic population regime', i.e. at larger ϕ (small $\phi_c - \phi$). In fact, the very same description of the dynamics in terms of x (the fraction of volume occupied by the largest droplet) is expected to be valid as the CPI is approached (or, anyway, in the regime of close-packing), as we underline in the text ("We posit that, at approaching CPI, this "kinetic"-level description might be further simplified, looking just at the dynamics of the volume $M(t)$ of the largest droplet"). In order to strengthen these ideas, we have added the following comment in the revised version:

On the other hand, deviations from the power-law scaling at large $\phi_c - \phi$, i.e. far from the CPI, are not surprising, since scaling behaviour is only to be expected near the critical point, even in standard equilibrium critical phenomena. Moreover, as we previously discussed, the very same approach in terms of the x variable should only be trusted as the CPI is approached, or, at least, in the close-packing regime.

Also, we introduce the definition of the Wiener process in the text.

Point 15: "Fig. 6: Indicate the volume fraction used in each panel."

Reply to Point 15: We thank the reviewer for the suggestion, which is now implemented.

---

[1] L. Yi, C. Wang, T. van Vuren, D. Lohse, F. Risso, F. Toschi, and C. Sun, Physical mechanisms for droplet size and effective viscosity asymmetries in turbulent emulsions, J. Fluid Mech. **951**, A39 (2022).

[2] M. Fruchart, R. Hanai, P. Littlewood, and V. Vitelli, Non-reciprocal phase transitions, Nature **592**, 363 (2021).

[3] M. J. Bowick, N. Fakhri, M. C. Marchetti, and S. Ramaswamy, Symmetry, thermodynamics, and topology in active matter, Physical Review X **12**, 010501 (2022).

[4] T. Markovich and T. C. Lubensky, Nonreciprocity and odd viscosity in chiral active fluids, Proceedings of the National Academy of Sciences **121**, e2219385121 (2024).

[5] F. Leal-Calderon, V. Schmitt, and J. Bibette, *Emulsion science: basic principles* (Springer Science & Business Media, 2007).

[6] G. Vaessen and H. Stein, The applicability of catastrophe theory to emulsion phase inversion, J. Colloid Interface Sci. **176**, 378 (1995).

[7] A. Perazzo, V. Preziosi, and S. Guido, Phase inversion emulsification: Current understanding and applications, Adv. Colloid Interface Sci. **222**, 581 (2015).

[8] K. Piela, G. Ooms, and J. Sengers, Phenomenological description of phase inversion, Phys. Rev. E **79**, 021403 (2009).

[9] D. Bakhuis, R. Ezeta, P. A. Bullee, A. Marin, D. Lohse, C. Sun, and S. G. Huisman, Catastrophic phase inversion in high-Reynolds-number turbulent Taylor-Couette flow, Phys. Rev. Lett. **126**, 064501 (2021).

[10] I. Girotto, A. Scagliarini, R. Benzi, and F. Toschi, Lagrangian statistics of concentrated emulsions, J. Fluid Mech. **986**, A33 (2024).

[11] C. Coulaloglou and L. Tavlarides, Description of interaction processes in agitated liquid-liquid dispersions, Chem. Eng. Sci. **32**, 1289 (1977).

[12] P. Krapivsky, S. Redner, and E. Ben-Naim, eds., *A kinetic view of Statistical Physics* (Cambridge University Press, 2010).

[13] S. Maaß and M. Kraume, Determination of breakage rates using single drop experiments, Chem. Eng. Sci. **70**, 146 (2012).

[14] F. Janoschek, F. Toschi, and J. Harting, Size distribution of particles in saturn's rings from aggregation and fragmentation, Proc. Natl. Acad. Sci. USA **112**, 9536 (2015).

[15] E. Villermaux, P. Marmottant, and J. Duplat, Ligament-mediated spray formation, Phys. Rev. Lett. **92**, 074501 (2004).

[16] E. Villermaux, Fragmentation, Annu. Rev. Fluid Mech. **39**, 419 (2007).

[17] S. Kooij, R. Sijs, M. Denn, E. Villermaux, and D. Bonn, What determines the drop size in sprays?, Phys. Rev. X **8**, 031019 (2018).

[18] G. I. Taylor, The viscosity of a fluid containing small drops of another fluid, Proceedings of the Royal Society of London. Series A, Containing papers of a mathematical and physical character **138**, 41 (1932).

[19] I. M. Krieger and T. J. Dougherty, A mechanism for non-newtonian flow in suspensions of rigid spheres, Trans. Soc. Rheol **3**, 137 (1959).

[20] R. Pal, Novel viscosity equations for emulsions of two immiscible liquids, Journal of Rheology **45**, 509 (2001).

[21] F. Ravelet, R. Delfos, and J. Westerweel, Experimental studies of liquid-liquid dispersion in a turbulent shear flow, Advances in Turbulence XI , 331 (2007).

REPLY TO REVIEWER #3

The authors have carefully addressed my previous comments, revising the figures and expanding the text and discussion accordingly. In my assessment, the manuscript now meets the quality standards of the journal, and I therefore recommend it for publication.

We thank the reviewer for the positive recommendation to publish our manuscript.

REPLY TO REFEREE #5

The topic covered in this article is timely and offers some interesting insights into the catastrophic inversion during emulsification process. The authors have partly addressed the reviewers main concerns and the manuscript could suitable for publication after considering and including the following items.

First of all we would like to thank the reviewer for positive and constructive comments on our manuscript, in particular for considering it interesting and timely and suitable for Nature Communications. Here we address in details the last remaining concerns of the reviewer and are confident that these helped us further improving our manuscript for a broader audience.

Point 1: *The main conclusion is that coalescence and breakup of droplets play a major role in the kinetic stability of the system. Now, this main conclusion is a little vague since this is something widely known in the literature and offer no real insight. I strongly recommend the authors to eliminate it or rephrase it from both the abstract and conclusions.*

Reply to Point 1: We thank the reviewer for raising this point that helped us further to clarify this important aspect. While we reiterated the general picture of emulsions experiencing coalescence and breakup in the conclusions ("A large number of systems in nature are made of elementary entities which aggregate and break up under the influence of external forces such as, for example, hydrodynamic drag"), we never intended to mention this as being the *the main conclusion* of our work. Instead, we specified that

"in this work we introduced a theoretical framework that allows capturing the key phenomenology for very large concentrations, where the dynamics of coalescence and breakup is particularly active, and we show that the macroscopic rheological properties of the system are dynamically and precisely determined by the population dynamics between droplets of different sizes".

Furthermore, we say that

"our study offers a solid statistical physics framework to explain the basic physics beyond CPI in emulsions, that -more generally- embraces all those systems undergoing a phase transitions characterised by broken detailed balance of small-scale aggregation-fragmentation processes".

In other words, of course, we do not claim the (re)discovery of breakups and coalescence in flowing emulsions. We underline, on the other hand, that:

i) the rate of occurrence of breakups/coalescence increases dramatically with the volume fraction of the disperse phase, thereby allowing us to distinguish between two dynamic regimes: the static population regime (characterised by a low rate of breakups/coalescences) and the dynamic population regime (high breakup/coalescence rates);

ii) at approaching the critical volume fraction for the catastrophic phase inversion, the mechanisms of breakup and coalescence are not symmetrical and are characterized by different timescales.

iii) a quantitative model helps to capture the observed phenomenology of this ssystem and, more generally, could be the basis for describing other systems influenced by aggregation and breakup processes.

In order to better convey this message and following the Referee's suggestion, in the Abstract we have rephrased the paragraph starting with "Our study clearly highlights..." as follows:

Our study clearly highlights that at high volume fractions, the droplet population starts to fluctuate wildly, leading to dramatic changes in the emulsion's rheology and stability.,

and we have fully revised the Conclusions as follows:

We have presented a comprehensive study combining experiments, numerical simulations, and theoretical modelling, aimed at characterizing the structural and morphological evolution of flowing stabilized emulsions as the volume fraction of the dispersed phase is increased up to the critical point where catastrophic phase inversion occurs. Two different flow conditions

have been considered: a Taylor–Couette setup (experiments) and large-scale homogeneous and isotropic forcing (simulations).

We showed that below a volume fraction of $\approx 65\%$ (roughly corresponding to the random close packing of spheres in three dimensions), the droplet number and typical sizes do not fluctuate significantly; correspondingly, the effective viscosity of the system closely follows standard phenomenological correlations with the volume fraction (i.e., the Krieger–Dougherty law). Conversely, above 65%, strong deviations arise (attributable to the emergence of extremely non-Newtonian rheology), along with a markedly dynamic structural evolution in which breakup and coalescence play a key dynamical role, with their rates increasing dramatically [**?**].

Focusing on how the rheology changes as the system approaches catastrophic phase inversion, we introduced a novel theoretical framework characterized by the presence of an order parameter—the size of the largest droplet—capable of bridging structural evolution and rheology through a common critical dynamics, appearing as a power-law divergence of fluctuations.

Furthermore, we proposed a dynamical model able to reproduce the critical behaviour of fluctuations of the order parameter while simultaneously highlighting the role of asymmetry and mismatched time scales between breakup and coalescence processes, revealing the close connection of catastrophic phase inversion with non-reciprocal phase transitions.

Point 2: *The article is presented in the introduction as dealing with the emulsification process while in the conclusion mention that the objective is to study stability after formation "As a specific case, we focus on stabilized emulsions of immiscible fluids where the aggregation process consists in droplet coalescence and the breakup of droplets into smaller droplets". Since you are mentioning breakup of droplets, it is clear that you are dealing with emulsion formation. The way the article is written makes very difficult to fully understand the main objective given these contradictory statements and this must be fully revised.*

Reply to Point 2: We thank the Referee for this important remark. It motivated us to carefully reread the manuscript and we acknowledge that, indeed, the term 'emulsification' was employed sometimes in a way that could have confused a reader. The study deals, in fact, with the stability of emulsions *under flow*, following a protocol (both in experiments

and simulations) that can be schematically thought of as consisting of two parts: i) a first phase (the actual *emulsification*, strictly speaking), whereby the dispersed phase is slowly added *while stirring*, until the desired volume fraction is reached; ii) a second phase, during which we continue to stir the emulsion at fixed volume fraction, while probing its rheology (torque measurements) and structure (droplet sizes). This is shown in Figs. 1 and 2. In both phases, in general, *both* breakup and coalescence events occur. The emulsification phase, though, by construction, is not a stationary process and, therefore, breakups and coalescence can outnumber each number (breakups presumably will prevail at lower volume fractions); instead, during the second phase (let us call it *stirring phase*) the system reaches a statistically stationary state, characterized by a dynamical equilibrium whereby breakups and coalescences, continuously occurring, on average balance each other. As the volume fraction increases, the rates of both types of events in the stirring phase grows; eventually, the balance is broken and catastrophic phase inversion takes place. Since we essentially focus on the latter statistically stationary phase, in the revised version we get rid of the term 'emulsification', when misleading and/or inappropriate: in the Introduction we say

Even more importantly, the complex flow environment (turbulent or chaotic) under which CPI in stirred emulsions takes place does not enter in the description;

the second section has been renamed

Stirring emulsions at high volume fraction;

finally, we rephrased the incipit of the caption of figure 10 as

A graphical visualization of the temporal evolution of the cumulative distribution $\overline{n}_I(t) = \sum_{i \in I} n_i(t)$ for a few representative size-classes of droplets during a typical run from fully resolved numerical simulations at high volume fraction.

Moreover, we explicitly talk of

flowing emulsions

wherever it might otherwise lead to misunderstandings.

Point 3: *One of the reviewers commented that the organization of the article is not adequate and I think that there is room for improvement in the revised version. For example, the introduction is unnecessarily complex and describes aggregating systems at the beginning to slowly moving to the main topic which is emulsion stability. Also, there is a mistake when saying that "...prone to phase inversion, namely a microstructural, irreversible, change towards the thermodynamically favoured configuration, whereby the continuous minority phase becomes disperse". This is simply not true. The final thermodynamic stable configuration is phase separation under the conditions described. Please, rephrase this comment.*

Reply to Point 3: We apologize if the Introduction appeared deviating from the article topic; we do not think that it is too complex to read though. We remark that the main topic of our work is not "emulsion stability" in general terms (which may mean many things and depend on many factors), but a specific instance of the stability of *flowing* emulsions, namely catastrophic phase inversion. This said, we deemed appropriate to the targeted journal (Nature Communication) an introduction where we drew possible analogies with different systems, governed by similar dynamics, in order to stress the generality of the proposed theoretical approach. So, following the Referee's comment, yet trying to convey such message, we have rephrased and shortened the first part of the introduction as follows:

Many natural systems, made of basic entities suspended in a flowing fluid, display the tendency of forming dynamically evolving aggregates. The fluid flow plays a dual role: it can considerably accelerate the aggregation process and, at the same time, be responsible for the breakup of larger aggregates. At a critical concentration, this aggregation/breakup balance is broken and the system undergoes a phase transition with the formation of a single integral-scale aggregate. Transitions of this kind include...

Concerning the second point, our definition (*"the continuous minority phase becomes dis-*

perse") is very common in the literature on catastrophic phase inversion. We remark, in fact, that in presence of a stabilisation mechanism (which is, indeed, present in the experiments and modelled in the numerical simulations, mimicking the effect of surfactants - see also the answer to Point 5), the full phase separation mentioned by the Referee is prevented.

Point 4: *While I fully understand that the authors are trying to shed light on the process from a physical point of view, there are some important aspects for researchers working on emulsion stability and formulation that are not present. One is the real size of the droplets. The results showed are normalized by $\langle D_0 \rangle$ but no mention to that value (or I was not able to find it). And the second is the real polydispersity percentage of the emulsions obtained in the experiments. These two variables are key to determine the stability of the system alongside the volume fraction. Depending on the droplet size, Brownian fluctuation, Ostwald ripening, hydrodynamic interaction, and droplet deformability plays different roles on the fate of the emulsion. The authors must discuss these aspects and how they consider their results can be framed.*

Reply to Point 4: We thank the Referee for spotting this omission, which we apologize for. The value is $D_0 \approx 200\,\mu$m. The polydispersity fluctuates in time and varies as a function of the volume fraction, as evidenced in Fig. 5 where we show the standard deviation of the diameter, which can be considered as a proxy of the polydispersity. Far from the phase inversion, the experimental value is $\sigma_D \approx 10^{-2} D_0 \approx 2\,\mu$m. We have added this information in the Materials and Methods, section A, and in the caption of Figure 9, writing as follows:

The value of D_0 in the experiment is $D_0 \approx 200\,\mu$m, whereas in the simulation it is $D_0 \approx 25\,$l.u. (lattice units).

Finally, we would like to underline again that, here, we always refer to the stability of *flowing* emulsion against *catastrophic phase inversion*, and not to the stability of *static* emulsion against *phase separation* of the two liquids; therefore, mechanisms such as creaming, Ostwald ripening, etc, are not expected to be relevant, for occurring on time scales much

longer than that characteristic of the flow. For instance, in the case of the Ostwald ripening (or, better, of the coarsening process, in the high volume fraction case), the characteristic time can be estimated as $t_{\rm coarse} \sim d/D_{\rm eff}$ [?], where d is the typical droplet size (e.g. mean diameter) and $D_{\rm eff}$ is an effective diffusion coefficient; the typical time of droplet dynamics due to the flow, instead, can be estimated as $t_{\rm adv} \sim d/U_{\rm rms}$. The ratio of these two times, $t_{\rm coarse}/t_{\rm adv} \sim U_{\rm rms} d/D_{\rm eff}$ can be interpreted as an *effective* Peclet number and it is typically very large (in the simulations, for instance, we estimate Pe ~ 2500). Moreover, given the typical size of the droplets, our emulsions can be considered "athermal", i.e. thermal fluctuations effects are negligible, in fact $\sqrt{k_B T/\gamma}/\langle D \rangle \sim \mathcal{O}(10^{-6})$; we added the following comment in the Materials and Methods, section B:

We remark that a fully hydrodynamic approach of this kind is justified here since we aim to describe emulsions made of droplets of sizes $d \sim 10^2\,\mu$m (see previous section), such that thermal fluctuations may be neglected, in fact, at room temperature $T \approx 300\,$K, $\sqrt{k_B T/\gamma}/d \sim \mathcal{O}(10^{-6})$.

Point 5: *The absence of surfactant is fully understandable give the complexity it adds in both experiments and simulations. However, this absence strongly limits the impact of the work and the potential application in more realistic emulsions. The fitting parameters partly contains these pieces of information and has been used in different theories to account for interfacial rheology. The authors can also discuss this aspect and frame their findings accordingly.*

Reply to Point 5: The Referee is right in pointing out the importance of surfactant in the physics of concentrated emulsions. The main role of surfactants, in fact, is to effectively lower the surface tension, thus inhibiting droplet coalescence and reduction of the total interface. Although we do not add/model explicitely surfactants in our experiments/simulations, we do have mechanisms that stabilize the interfaces and prevent full phase separation. This information is provided in the Materials and Methods, sections A and B. In the experiments, surface-active impurities accumulated at the oil–water interfaces determines the stabilization, although some differences exist with the surfactant-stabilized

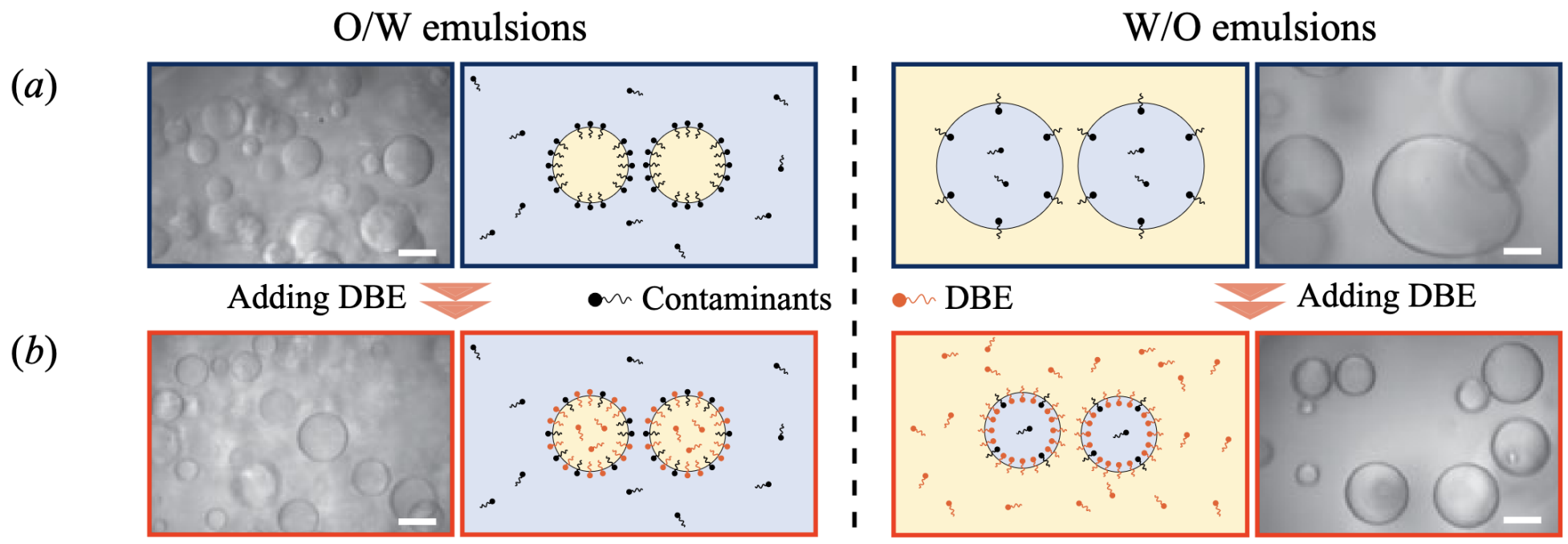

FIG. 1: (a,b) Schematic diagrams and microscopic images of the effects of surfactants (or surface-active contaminants) on the emulsions. Panel (a) depicts the emulsions without adding surfactant. The asymmetric behaviours of the droplet size between O/W emulsions and W/O emulsions are observed due to the difference in the concentrations of contaminants on the interfaces. The deformation of the droplet is observed clearly in W/O emulsions. Panel (b) represents emulsions with added DBE at 250 μL L^{-1}. The surfactant is adsorbed into the interface at saturation for both O/W and W/O emulsions. The symmetry of droplet size is almost recovered. Snapshots of the emulsion at an oil volume fraction $\phi_O = 5\%$ are used to represent O/W emulsions, while W/O emulsions are denoted by snapshots of the emulsion at $\phi_O = 95\%$. For all snapshots here, $Re = 5.2 \times 10^3$. The scale bar represents 200 μm. Figure taken from L. Yi et al, *J. Fluid Mech.* **951**, A39 (2022).

dispersion, e.g. the droplet size asymmetry betweem O/W and W/O emulsions (see Reference [?] and Fig. 1). In the simulations the stabilization is achieved through properly devised competing lattice interactions that promote a positive disjoining pressure in liquid films separating two close interfaces (see, e.g., Reference [?] and Fig. 2). We have commented on this aspect in the main text (section "Emulsification at high volume fraction") of the revised version as follows:

To achieve a concentrated (densely packed) emulsion of droplets (e.g. “oil-in-water” or O/W emulsion), a stabilisation of the interface that inhibits coalescence is needed: in our experiments this is provided by surface-active impurities accumulated at the oil–water interfaces [?], whereas in the simulations it is achieved through properly devised competing lattice interactions [? ?]. Moreover, it is necessary to slowly add the dispersed phase

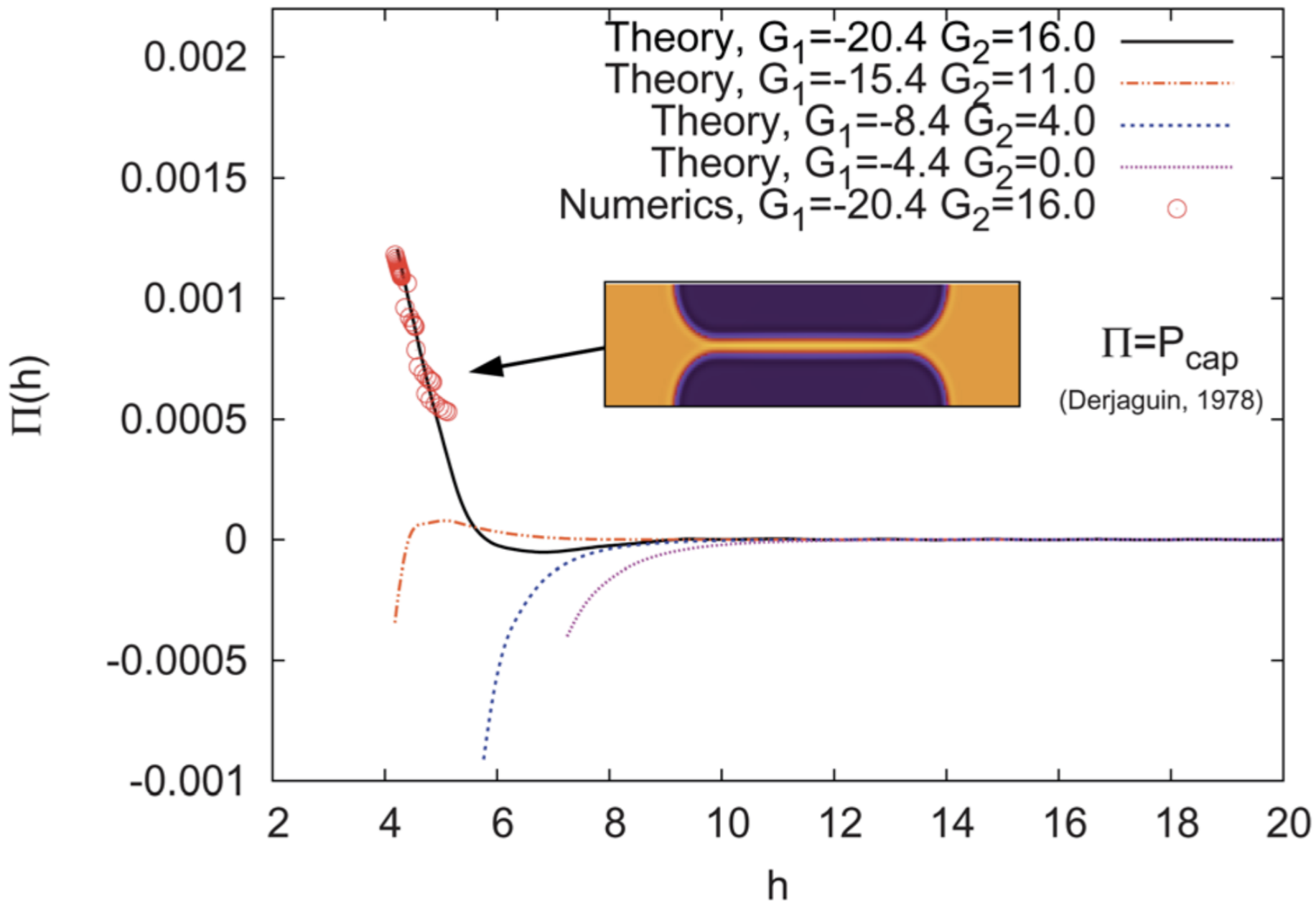

FIG. 2: A direct measurement of the disjoining pressure. Two hemispherical bubbles are faced against each other and form a thin liquid film between them (a $2d$ case is shown, for simplicity). The film is stabilized against coalescence by a suitable choice of the parameters corresponding to a positive disjoining pressure. The mechanical definition of Derjaguin and Churaev (B.V. Derjaguin and N. Churaev, *J. Colloid Interface Sci.* **66**, 389–398 (1978)) is then applied and the disjoining pressure is measured from the capillary pressure at the curved interface. Different theoretical predictions are shown for different values of the coupling parameters at fixed bulk pressure (see [**?**] for further theoretical details). Figure taken from M. Sbragaglia et al, *Soft Matter* **8**, 10773 (2012).

in order to maintain the system in an intrinsically metastable state, i.e. energetically less favored with respect to the inverted emulsion (i.e. “water-in-oil”, W/O). Further details on how this is realized in the experiments and in the simulations are given in the Materials and Methods section.

Reviewer #1
(Remarks to the Author)

This manuscript investigates the coalescence breakup dynamics of concentrated emulsions approaching catastrophic phase inversion (CPI) through a combined experimental, numerical, and theoretical study. The authors propose a reduced dynamical model where the fraction of volume occupied by the largest droplet acts as an order parameter. They report that the loss of detailed balance between breakup and coalescence leads to non-reciprocal dynamics and critical fluctuations near CPI. The topic is timely and relevant to soft matter and non-equilibrium systems, and the paper is clearly written and well organized. The combination of Taylor-Couette experiments, direct numerical simulations, and a reduced stochastic model is a significant strength. The close correspondence between experimental observations and simulation data convincingly supports the proposed framework. The idea of describing CPI using a non-reciprocal dynamical-system perspective is novel and potentially impactful. However, before the manuscript can be considered for publication, I recommend the following clarifications and improvements:

1. The derivation and physical interpretation of the stochastic model (Eqs. 1–2) should be discussed in more detail. How are the parameters α, r, β, ε_0, and ε_1 related to measurable quantities such as coalescence or breakup rates?
2. The experimental section should include the type and concentration of surfactant, interfacial tension, and relevant Reynolds/Weber numbers. These are essential for reproducibility and for interpreting the reported critical volume fraction.
3. The scaling of fluctuations $\sigma \sim (\phi c - \phi)^{-1/2}$ is intriguing but should be statistically supported. Please provide uncertainty estimates or fit confidence intervals.
4. The term “non-reciprocal phase transition” could be explained more clearly. A short comparison with recent literature on non-Hermitian or active-matter transitions would help situate the work.
5. The introduction could be condensed by removing background repetition and focusing more sharply on how this study advances understanding beyond classical population-balance or catastrophe-theory descriptions.
6. The model assumes that breakup and coalescence can be reduced to two scalar rates (α, f). This simplification implicitly neglects droplet–droplet hydrodynamic coupling and shape anisotropy near jamming. The authors should justify why this reduction still captures the observed hysteresis and bimodality, or show that including higher-order coupling terms does not change the main conclusions.
7. The DNS comparison relies on a diffuse-interface approach, yet the interface width correction is mentioned only qualitatively. Quantify how sensitive the predicted critical volume fraction (ϕc) is to interface resolution and to the choice of surface-tension parameters.

Review report on Article Paper
–
"Non-reciprocal coalescence-breakup dynamics in concentrated emulsions"

by Ivan Girotto, Andrea Scagliarini, Lei Yi, Roberto Benzi, Chao Sun, and Federico Toschi.

Nature Communications, manuscript APS/123-QED

The manuscript investigates catastrophic phase inversion (CPI) in dense stabilized emulsions through a combination of Taylor–Couette experiments, direct numerical simulations, and a reduced dynamical-systems model. The authors argue that close to the inversion threshold, the emulsion dynamics can be captured by a single coarse-grained variable, specifically the size of the largest droplet, which acts as a dynamical order parameter. They report a transition from Gaussian to bimodal fluctuations in both effective viscosity and droplet-size statistics, interpreted as signatures of a non-reciprocal dynamical phase transition governed by competing coalescence and breakup processes.

Strengths:
The topic is timely, and understanding CPI is of clear relevance for soft-matter physics, rheology, emulsion formulation, and consequently many industrial applications such as food processing or care products. The combination of experiments, advanced simulations, and minimal modeling is appealing. Both the scientific topic and methods are potentially a good fit for the broad audience of *Nature Communications*.

Specific Issues, Weaknesses, and Recommendations:
However, in its current form, the manuscript falls short in several aspects, making it hard to recommend for publication: it suffers mostly from presentation and organizational issues, as well as from incomplete or unclear explanations: many claims are made without sufficient demonstration or (quantitative) support. *Major revisions are needed.* My comments and suggestions are listed below, including both major points and more specific remarks following the chronological order of the manuscript.

1. One of my major points concerns the structure of the article: the authors

present experiments and then attempt to interpret them with advanced simulations. As it stands, experimental results are introduced with minimal detail in the main text (relying on the Methods section), which leads to two problems: (i) the experimental results are difficult to follow and understand, and (ii) the connection to the simulations, which are meant to interpret underlying mechanisms, is unclear.

(a) I suggest merging Figures 1 and 2 to provide a single integrated view of the experimental findings. This merged figure should include the experimental setup, temporal evolution of key metrics (largest droplet size, rms velocity, effective viscosity, etc.), and representative snapshots showing morphological changes over time. Including the experimental sketch from Fig. 8(a) would also help clarify the setup. This integration will make the paragraph starting around line 78 more readable.

(b) Fig. 1(b): The emulsion morphology change is not visually clear. Adjust contrast or add annotations to make droplet size and distribution changes more obvious.

2. Lines 35-40: The discussion of external forces driving aggregation and fragmentation is useful, but I am surprised there is no mention of droplet formation (e.g., sprays) or general particle aggregation, with reference to canonical aggregation theory (Smoluchowski). Including these references would strengthen the context.

3. Line 88: While further details are provided in the Methods section, the main text must provide essential experimental information. In particular, the relation between injected volume and volume fraction (Eq. 3) should be clarified: it is non-trivial and important for reproducibility.

4. Line 93: The statement that "the population is relatively static" should be directly demonstrated in the figures. Merging Fig. 1 and 2 would help clarify how both experiments and simulations evolve over time.

5. Page 5, Fig. 1(a): This figure could be made more quantitative. Many empirical or theoretical models describe the dependence of effective viscosity on oil concentration (dilute, semi-dilute, concentrated regimes). A comparison to such models would be informative; authors knows a priori (figure 9 in the supplementary materials what is the droplet size dependence with the oil concentration). Additionally, the manuscript should discuss reproducibility: is only one experiment shown? Since conclusions rely on statistical distributions, reproducibility is essential.

6. Line 98: "Through time-resolved global torque"—this must be demonstrated in the main text. Relevant information from the Methods/Supplementary could be summarized here.

7. Lines 104-107: Statements regarding "significant droplet deformation" and "droplet size" must be visually supported.

8. Line 112: Droplet "lifetime" is introduced abruptly without prior definition. This paragraph is unclear and should be rewritten to convey a structured message.

9. Lines 116, 124-125: Reynolds and Weber numbers should be defined when first introduced.

10. Fig. 2: Consider adding a vertical axis indicating increasing oil concentration. Define the right-axis units (speed) and clarify whether the rms speed refers to the largest droplet, all droplets, or some other measure (centroid, center-of-mass?). The rationale for focusing on the largest droplet should also be explicitly stated in the text.

11. Line 168: Results in Figures 2 and 4 are compared verbally but not directly or plotted together. Consider plotting them together for clarity.

12. Line 175: "CPI is transition is appealing"—please rephrase for clarity.

13. Figures 3 and 4: Consider merging these figures for clarity. Explicitly state whether time is adimensionalized. Provide the time step between points in Fig. 4.

14. Lines 217-220: The discussion of characteristic times (Wiener processes?) is unclear. Provide context and connect explicitly to the dynamics shown in figures.

15. Can the authors comment on deviations at large $\phi - \phi_c$?

16. Fig. 6: Indicate the volume fraction used in each panel.
